# Enhancing the Photosensitivity of Hypocrellin A by Perylene Diimide Metallacage-Based Host–Guest Complexation for Photodynamic Therapy

**DOI:** 10.1007/s40820-024-01438-w

**Published:** 2024-06-25

**Authors:** Rongrong Li, Tianfeng Yang, Xiuhong Peng, Qian Feng, Yali Hou, Jiao Zhu, Dake Chu, Xianglong Duan, Yanming Zhang, Mingming Zhang

**Affiliations:** 1 State Key Laboratory for Mechanical Behavior of Materials, Shaanxi International Research Center for Soft Matter, School of Materials Science and Engineering, Xi’an Jiaotong University, Xi’an, 710049 People’s Republic of China; 2 School of Pharmacy, Health Science Center, Xi’an Jiaotong University, Xi’an, 710061 People’s Republic of China; 3 Department of Rehabilitation Medicine, Shaanxi Provincial People’s Hospital, Xi’an, 710068 Shaanxi People’s Republic of China; 4 Department of Gastroenterology, The First Affiliated Hospital of Xi’an Jiaotong University, Xi’an, 710061 People’s Republic of China

**Keywords:** Metallacages, Host–guest interactions, Fluorescence resonance energy transfer, Singlet oxygen, Photodynamic therapy

## Abstract

The development of supramolecular hosts which can efficiently encapsulate photosensitizers to improve the photodynamic efficacy holds great promise for cancer therapy. Here, we report two perylene diimide-based metallacages that can form stable host–guest complexes with planar conjugated molecules including polycyclic aromatic hydrocarbons and photosensitizers (hypocrellin A). Such host–guest complexation not only prevents the aggregation of photosensitizers in aqueous environments, but also offers fluorescence resonance energy transfer (FRET) from the metallacage to the photosensitizers to further improve the singlet oxygen generation (Φ_Δ_ = 0.66). The complexes are further assembled with amphiphilic polymers, forming nanoparticles with improved stability for anticancer study. Both in vitro and in vivo studies indicate that the nanoparticles display excellent anticancer activities upon light irradiation, showing great potential for cancer photodynamic therapy. This study provides a straightforward and effective approach for enhancing the photosensitivity of conventional photosensitizers via host–guest complexation-based FRET, which will open a new avenue for host–guest chemistry-based supramolecular theranostics.

**Supplementary Information:**

The online version contains supplementary material available at 10.1007/s40820-024-01438-w.

## Highlights

- A new type of metallacage was successfully fabricated and used as containers for hypocrellin-type photosensitizers, which prevented the self-aggregation of photosensitizers in aqueous solution.
- The metallacage was also employed as an energy donor to promote the singlet oxygen generation ability via fluorescence resonance energy transfer, thereby achieving highly efficient photodynamic therapy.

**Supplementary Information:**

The online version contains supplementary material available at 10.1007/s40820-024-01438-w.

## Graphical abstract

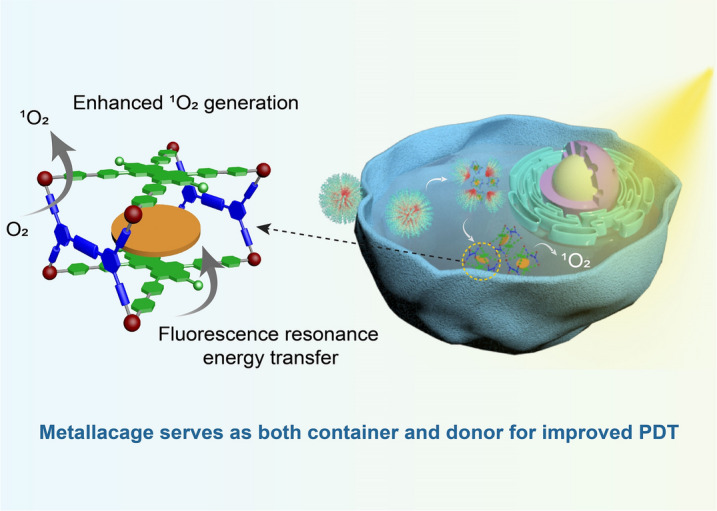

## Introduction

Although chemotherapy is still the most widely used approach in the treatment of tumors, it often suffers from severe side effects, non-targeting capability, and potential drug resistance, which greatly limits its further applications in clinical trials [1–3]. In order to solve this problem, combinational therapy is developed via the reasonable integration of other therapeutic methods to increase the efficacy and reduce the side effect of cancer treatment [4–7]. In this regard, photodynamic therapy (PDT) has been considered to be a promising complementary strategy to chemotherapy because of its negligible drug resistance, minimal invasion, fewer side effects, and less damage to normal tissues [8–13]. However, conventional photosensitizers used in PDT are generally large π-conjugated planer small molecules such as porphyrin and hypocrellin derivatives [14–17]. Such molecules are easy to aggregate in aqueous solution due to π–π stacking and hydrophobic interactions, leading to the quench of the excited state and poor singlet oxygen (^1^O_2_) generation quantum yield, which significantly impedes their broader applications. The encapsulation of photosensitizers in supramolecular hosts not only prevents their aggregation and promotes the generation of ^1^O_2_, but also offers possibilities for introducing extra functionalities such as targeting ligands, imaging agents, or therapeutic drugs for supramolecular photochemotherapy [18–20]. Moreover, the stimuli-responsiveness of supramolecular systems may also provide controllable release of the agents. Therefore, the development of efficient supramolecular hosts for photosensitizers and the exploration of their applications in cancer photochemotherapy are demanding.

**Scheme 1:**
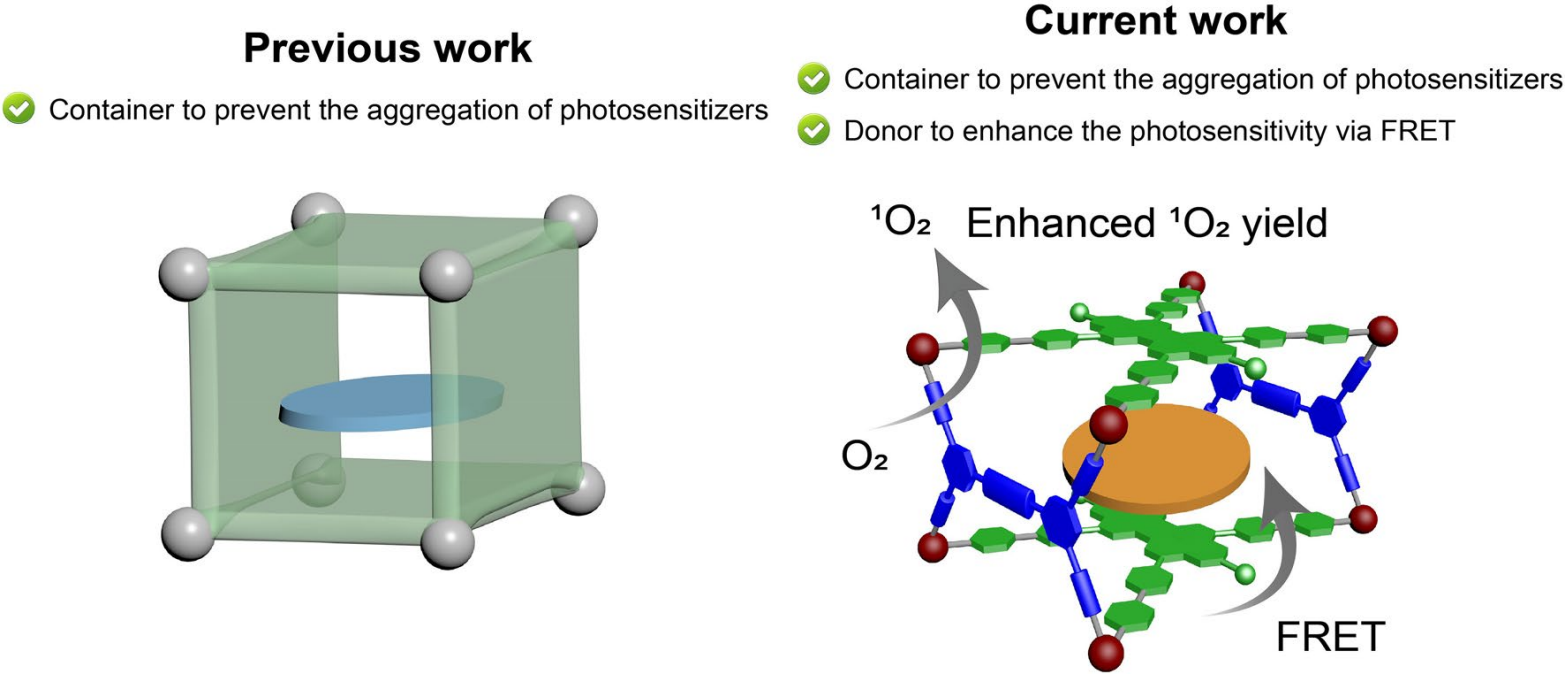
Cartoon illustrations of the role of metallacages in photodynamic therapy

Metal–organic cages or metallacages are a type of three-dimensional supramolecular assemblies formed by metal-coordination bonds [21–29]. They generally possess well-defined cavities capable of encapsulating guest molecules, enabling their wide applications in chemical sensing and recognition [30–34], adsorption and separation [35–41], catalysis [42–46], etc. Recently, their application as supramolecular containers for the delivery of photosensitizers for PDT has received much attention due to their convenient preparation, tunable structures, and ease of functionalization [47–50]. For example, Therrien et al. reported the preparation of prismatic metallacages to deliver and spatial-controlled release porphin for PDT [51]. Mukherjee et al. [52] described a water-soluble barrel-shaped metallacage to encapsulate zinc-phthalocyanine for enchanced PDT. Besides preventing the aggregation of their encapsulated photosensitizers, they can also serve as a type of therapeutic drugs based on the inherent cytotoxicity of metal nodes, increasing the efficacy of cancer treatment via the combination of photo and chemotherapy [53, 54]. However, in most cases, the metallacages only served as containers (Scheme 1); their use as energy donors to promote the ^1^O_2_ generation ability of the encapsulated photosensitizers and thus increase the performance of PDT has been rarely addressed. Such study will not only benefit for the rational design and preparation of functional metallacages toward real applications, but also effectively integrate the advantages of both components for improved synergistic therapy, which will promote the development of metallacage-based supramolecular biomaterials.

Herein, we report a type of perylene diimide (PDI)-based emissive metallacages which serves as both hosts and energy donors to promote the ^1^O_2_ generation ability of their encapsulated photosensitizers via fluorescence resonance energy transfer (FRET) [55–57] and then use the supramolecular system for improved cancer photochemotherapy. The metallacages are prepared via the multicomponent coordination-driven self-assembly of a tetracarboxylic ligand (**1**), *cis*-(PEt_3_)_2_Pt(OTf)_2_ (**2**), and tetrapyridyl perylene diimide (**3a** or **3b**). The distance between the two PDI planes can be finely adjusted via the regulation of PDI ligands, offering **Cage 4b** as a more efficient host for photosensitizer hypocrellin A (**G**_**5**_) (*K*_*a*_ = (1.51 ± 0.04) × 10^5^ M^−1^ in CH_3_CN/H_2_O). Owing to the good spectral overlap between the emission of the metallacage (**Cage 4b**) and the absorption of the photosensitizer (**G**_**5**_), efficient FRET takes place from **Cage 4b** to **G**_**5**_, thus resulting in enhanced ^1^O_2_ generation quantum yields. It is worth mentioning that the ^1^O_2_ quantum yield (Φ_Δ_) of complex **Cage 4b** ⊃ **G**_**5**_ reaches 0.66, which is 1.67 times that of the photosensitizer alone. Complex **Cage 4b** ⊃ **G**_**5**_ is further assembled with amphiphilic *m*PEG-DSPE to form nanoparticles (**NPs 5**) for cancer photodynamic therapy, showing extraordinarily high inhibition rate toward MHCC-97L cells (liver cancer) and NCI-H460 cells (lung cancer). **NPs 5** displays the most substantial antitumor effectiveness, demonstrating the highest inhibition of tumor growth in 14 days after the treatment, affirmed by the smallest tumor volume among all the experimental groups. In contrast, the control group treated by **3b**, **G**_**5**_, **Cage 4b**, or **Cage 4b** ⊃ **G**_**5**_, exhibits significantly lower anticancer activity compared to **NPs 5**, which is in good agreement with the tumor mass results. This outcome reveals that **NPs 5** exhibits the best photodynamic activity for MHCC-97L tumor-bearing nude mice compared with the sole **Cage 4b**, photosensitizer **G**_**5**_ and other controlled compounds **2** and **3b**, consistent with the high ^1^O_2_ generation efficiency of complex **Cage 4b** ⊃ **G**_**5**_. Furthermore, in vivo anticancer studies reveal that **NPs 5** exhibits an augmented inhibitory effect on tumor proliferation. All these in vivo anticancer results indicate that the photosensitivity of the photosensitizers can be significantly enhanced through FRET process, leading to desirable performance for improved PDT. This study represents an effective strategy to employ host–guest interactions to enhance the ^1^O_2_ generation efficiency via FRET for cancer photochemotherapy, which will promote the biological applications of metallacages.

## Experimental and Methods

### Self-Assembly of Metallacages

Compounds **1**, **2,** and **3a** (or **3b**) in a 1:4:1 molar ratio were dissolved in acetonitrile (5 mL), and then, the reaction mixture was heated at 50 °C for 8 h. After being cooled to room temperature, the solvent was removed by nitrogen flow. The residue was redissolved in acetonitrile (1 mL) and filtered to give a clear solution, which was precipitated by the addition of diethyl ether to give **Cage 4a** or **Cage 4b** as a red powder.

### Growth of Single Crystals

Solutions of **Cage 4a** or **Cage 4b** in DMF (350 μL) with varying concentrations (4.0, 6.0, 8.0, 10.0, and 12.0 mg mL^−1^) were introduced into a 2-mL vial. The vial was then positioned within a 20-mL vial filled with toluene (5 mL). The entire system was left at room temperature for 1 month to get the crystals.

### Preparation of Nanoparticles

**Cage 4b** ⊃ **G**_**5**_ (3.25 mg) and *m*PEG-DSPE2000 (8 mg) were dissolved in acetone (1 mL) and pure water (5 mL), respectively, and then stirred for 10 min. The solution of **Cage 4b** ⊃ **G**_5_ was added into the solution of *m*PEG-DSPE2000 under sonication dropwisely. The mixture was then stirred at room temperature in dark for 36 h to remove acetone. The resulting solution was passed through a 0.2-μm syringe filter to give **NPs 5,** which was stored at room temperature for further use.

### Singlet Oxygen Quantum Yields Measurements

Singlet oxygen generation experiments were conducted in acetonitrile using a LED lamp with a wavelength of 520 nm as the light source. Prior to the experiment, the absorption of photosensitizers (including **3a**, **Cage 4a**, **3b**, **Cage 4b**, **G**_**5**_, **Cage 4a** ⊃ **G**_**5**_, and **Cage 4b** ⊃ **G**_**5**_) at the excitation wavelength of 520 nm was maintained at approximately 0.25. Quantum yields for singlet oxygen generation were determined by monitoring the reduction in the absorption of 1,3-diphenylisobenzofuran (DPBF) at 410 nm due to its photooxidation sensitized by the photosensitizers. Time-dependent absorption spectra of DPBF were recorded every 1.0 min for the homogeneous phase. The quantum yields of singlet oxygen generation (^1^O_2_) were calculated using a relative method with optically matched solutions. The calculation involved comparing the decrease in the absorbance of DPBF sensitized by photosensitizers to that of rose bengal (*RB*) (Φ_Δ_ = 0.54), which served as a reference, based on the equation:

$$\Phi_{\Delta }^{S} = \Phi_{\Delta }^{RB} \frac{{m^{S} F^{RB} }}{{m^{RB} F^{S} }}$$

where superscripts “*S*” and “*RB*” denote the photosensitizers and RB, respectively, “Φ_Δ_” is the quantum yield of singlet oxygen, “*m*” is the slope of a plot with a difference in the change in the absorbance of DPBF (at 410 nm) with the irradiation time, and “*F*” is the absorption correction factor, which is given by *F* = 1–10^−OD^ (OD corresponds to the absorbance of the photosensitizer).

### Cell Culture

The NCI-H460 human lung cancer cell line was acquired from the Shanghai Institute of Cell Biology at the Chinese Academy of Sciences in Shanghai, China. The MHCC-97L (97L) HCC cell line was generously provided by the First Affiliated Hospital of Xi'an Jiaotong University. MHCC-97L cells and NCI-H460 cells were cultured in DMEM and RPMI 1640, respectively, supplemented with 10% fetal bovine serum (FBS) from ExCell Bio, China, and 100X penicillin/streptomycin (100 U mL^−1^ and 100 μg mL^−1^) from Solarbio, China, at 37 °C with 5% CO_2_.

### Confocal Laser Scanning Microscopy Imaging

MHCC-97L and NCI-H460 cells were seeded at a density of 1 × 10^4^ cells on a circular coverslip (diameter 12 mm) in complete DMEM culture medium. Following a 24-h incubation period, the cells were exposed to various compounds at a concentration of 5 μM for 15 min. Subsequently, the cells were rinsed twice with cold PBS. F-actin was stained using 4′,6-diamidino-2-phenylindole (DAPI) and visualized using a Zeiss LSM 710 confocal microscope. The data were analyzed using ImageJ. It was measured at 490 nm using a Bio-Rad 680 microplate reader.

### Intracellular Reactive Oxygen Species Production Study

Cells were seeded in a glass culture dish for 24 h, then continued to be cultured in fresh medium containing compounds (2 μM) for 8 h. Subsequently, the cells were exposed to 1 mL of fresh serum-free medium containing 10 μM DCFH-DA at 37 °C for 20 min. After thorough washing, the cells were subjected to white light treatment (50 mW cm^−2^) for 1 min, and flow cytometry analysis was conducted to examine intracellular reactive oxygen species production.

### MTT Assay

Cells were seeded in 96-well plates at a density of 5000 cells per well in 180 μL of complete medium and incubated in a 5% CO_2_ atmosphere at 37 °C for 24 h. Subsequently, the culture medium was replaced with 20 μL of freshly prepared culture medium containing compounds at various concentrations. The cells were further incubated for 48 h, after which the medium was substituted with fresh culture medium, and MTT solution (5 mg mL^−1^) was added. The cells were incubated for an additional 4 h to enable viable cells to reduce the yellow tetrazolium salt (MTT) into dark blue formazan crystals. Finally, 100 μL of lysis buffer was added to the wells and incubated for another 4 h at 37 °C. The absorbance was measured at 490 nm using a Bio-Rad 680 microplate reader.

### Cellular Staining

Cells were initially seeded in glass dishes for 24 h and subsequently subjected to four different treatments: (1) PBS solution; (2) PBS solution incubated with white light (50 mW cm^−2^) for 1 min; (3) media containing **NPs** **5** (2 μM) for 8 h without exposure to light; and (4) media containing **NPs** **5** (2 μM) for 8 h with illumination using white light (50 mW cm^−2^) for 1 min. Following the diverse treatments, cells were further incubated at 37 °C for 2 h and then stained with propidium iodide (PI) (60 μg mL^−1^) and fluorescein diacetate (FDA) (100 μg mL^−1^) in PBS for 10 min. Subsequently, the cells were meticulously washed and imaged by confocal laser scanning microscopy (CLSM), with excitation wavelengths of 488 nm for FDA and 543 nm for PI. The emission filters used were 500–550 nm for FDA and 550–650 nm for PI.

### In Vivo Tumor Therapy

All experimental protocols were approved by the Ethics Committee of the Xi'an Jiaotong University Health Science Center, Xi’an, China. The mice were randomly divided into seven groups (*n* = 5/group). To set up the tumor xenograft model, a total of 5 × 10^6^ MHCC-97L cells were subcutaneously injected into the right armpit of BALB/c female nude mice (4 weeks old) and permitted the tumor to reach a size over 100 mm^3^ in volume. Thirty-five tumor-bearing female nude mice were divided into seven groups (PBS group, *cis*-(PEt_3_)_2_Pt(OTf)_2_ group, **3b** group, **G**_**5**_ group, **Cage 4b** group, **Cage 4b** ⊃ **G**_**5**_ group, and **NPs** **5** group). These compounds were injected into the tumor at a final *cis*-(PEt_3_)_2_Pt(OTf)_2_ concentration of 2 mg kg^−1^, then treated with laser irradiation (520 nm, 50 mW cm^−2^ for 30 s). After 6-h post injection, the mice were exposed to Caliper IVIS Lumina II System to investigate the in vivo tracking of metallacages. Tumor size (*V* = *W*^2^ × *L*/2 mm^3^) was measured, and the body weight was recorded every 2 days for 14 days. At day 14, the tumors were collected and fixed in 4% paraformaldehyde overnight, embedded in paraffin, and sectioned at a thickness of 5 μm. The sections were stained with hematoxylin and eosin (H&E) or immunohistochemistry (IHC) of Ki 67.

### Statistical Analysis

All results are representative of data generated in each independent experiment. All numerical values were expressed as the mean ± SEM. For multiple comparisons, statistical analysis was performed using one-way ANOVA followed by a Bonferroni posttest. Data analysis was performed using SPSS 18.0 software and considered statistically significant at *p* < 0.05.

## Results and Discussion

### Preparation and Characterization of Metallacages

**Fig. 1:**
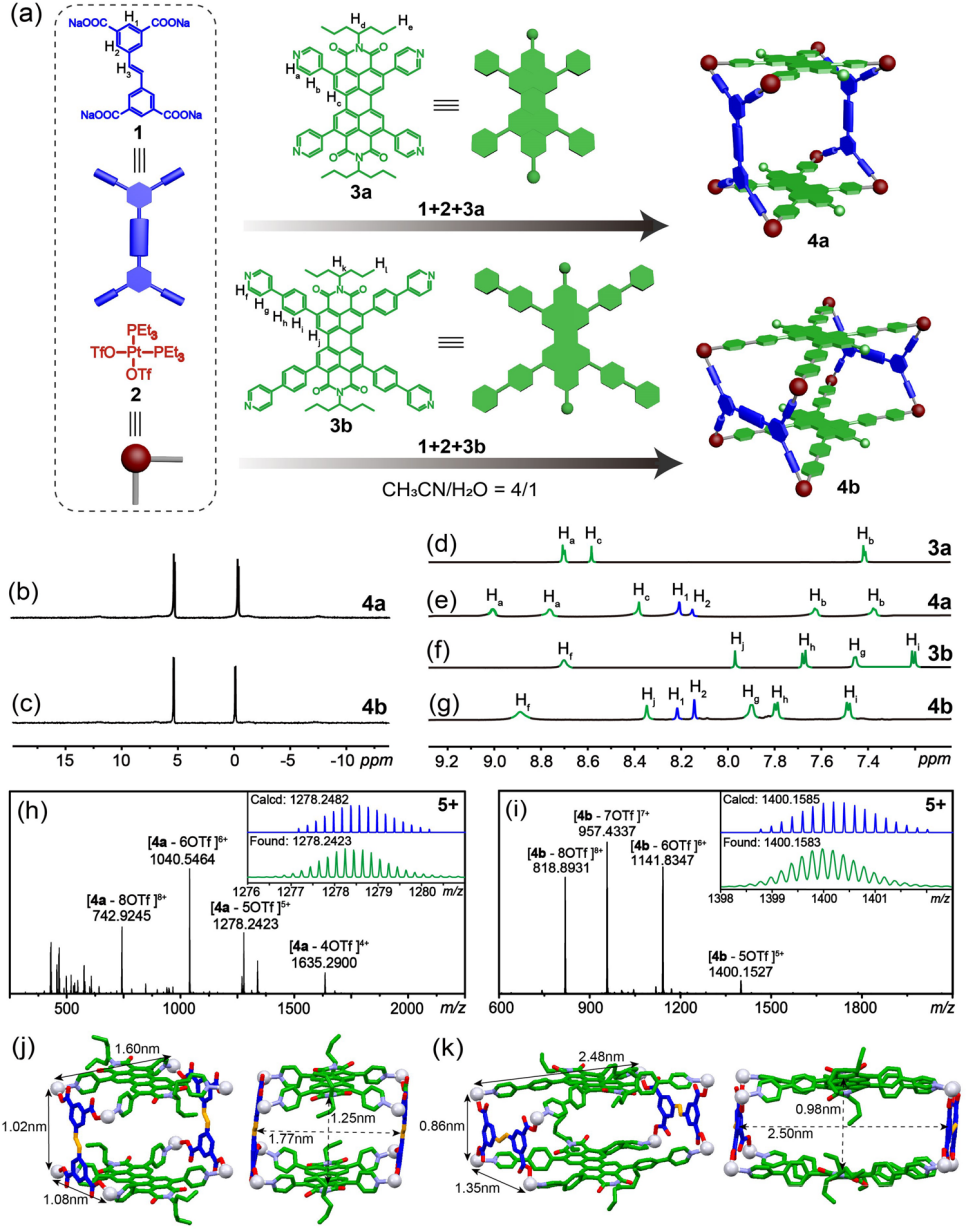
**a** Self-assembly of **Cage 4a** and **Cage 4b**. **b**, **c** Partial ^31^P{^1^H} NMR (150 MHz, CD_3_CN, 295 K) spectra of **Cage 4a** and **Cage 4b**. **d**–**g** Partial ^1^H NMR spectra (600 MHz, CD_3_CN, 295 K) of **3a**, **Cage 4a**, **3b **and **Cage 4b**. **h**, **i** ESI-TOF–MS spectra of **Cage 4a** and **Cage 4b**. **j** Crystal structures of **Cage 4a**. **k** Simulated structures of **Cage 4b**. Hydrogen atoms, triethylphosphine units, counterions, and solvent molecules were omitted for clarity

Metallacage (**Cage 4a** or **Cage 4b**) was prepared by the self-assembly of tetracarboxylic ligand (**1**), *cis*-(PEt_3_)_2_Pt(OTf)_2_ (**2**) and PDI ligand (**3a** or **3b**) in a 1:4:1 molar ratio (Fig. 1a, see Supporting Information of synthetic details). Their structures were confirmed by the combination of ^31^P{^1^H}, ^1^H NMR and electrospray ionization time-of-flight mass spectrometry (ESI-TOF–MS). The ^31^P{^1^H} NMR spectra exhibited two doublet peaks at 5.19 and − 0.47 ppm for **Cage 4a**, and 5.36 and − 0.12 ppm for **Cage 4b** (Fig. 1b, c). These doublet peaks, along with concurrent ^195^Pt satellites, indicated that each platinum atom coordinated with one pyridyl nitrogen of the PDI face and one carboxylic oxygen of the tetracarboxylic pillar. In the ^1^H NMR spectra, the *α*-pyridyl protons H_a_ and the *β*-pyridyl protons H_b_ of **Cage 4a** split into two sets of signals with noticeable chemical shifts (Fig. 1d, e), corresponding to protons inside and outside the cavity. For **Cage 4b**, only one set of protons (H_f_, H_g_, H_h_, H_i_ and H_j_) was observed, and all the protons exhibited obvious downfield chemical shifts (Fig. 1f, g), suggesting the formation of coordination bonds. The coordination stoichiometries of **Cage 4a** and **Cage 4b** were determined by ESI-TOF–MS (Fig. 1h, i). Isotopically well-resolved peaks with charge states ranging from 4 + to 8 + were observed for the two metallacages due to the loss of counterions (OTf^−^), confirming the composition of the metallacages. For example, peaks at *m*/*z* = 742.9245, 1040.5464, 1278.2423, 1635.2900, 818.8931, 957.4337, 1141.8347, and 1400.1527 were found, corresponding to [**Cage 4a** − 8OTf]^8+^, [**Cage 4a** − 6OTf]^6+^, [**Cage 4a** − 5OTf]^5+^, [**Cage 4a** − 4OTf]^4+^, [**Cage 4b** − 8OTf]^8+^, [**Cage 4b** − 7OTf]^7+^, [**Cage 4b** − 6OTf]^6+^, and [**Cage 4b** − 5OTf]^5+^, respectively. These data were entirely consistent with their calculated values. These findings align with previous reports [58–61], affirming the successful construction of the relevant discrete multicomponent metallacages.

To further elucidate the coordination structures of the metallacages, single crystals of **Cage 4a** suitable for X-ray diffraction analysis were successfully obtained through vapor diffusion of dioxane into DMF over 1 month. **Cage 4a** is composed of two tetrapyridyl PDI units and two tetracarboxyl panels connected by eight platinum(II) atoms, forming a barrel-shaped structure. The dimension of **Cage 4a** is 1.60 × 1.08 × 1.02 nm^3^, based on the distance between platinum atoms. The distance between the two parallel PDI faces is 1.25 nm, which is determined by the length of the tetracarboxylic ligands. As all the attempts to get the single crystals of **Cage 4b** failed, molecular simulation was conducted to unveil its coordination structure. The dimension of **Cage 4b** is 2.48 × 1.35 × 0.86 nm^3^. Because the distance between the neighboring pyridyl groups in **3b** is much longer than that in **3a**, tetracarboxylic ligand **1** is connected to the two neighboring pyridyl groups using its long side in **Cage 4b**, which has been also observed in our previous study [62]. The distance between the two PDI faces in **Cage 4b** is only 0.98 nm, much shorter than that in **Cage 4a**, which is better for the *π*–*π* stacking interactions between the guests and the metallacage hosts. Therefore, it is anticipated that both metallacages are capable of complexing planar guest molecules, while metallacage **Cage 4b** should exhibit stronger binding abilities with guests than **Cage 4a**.

### Host–Guest Complexation Study

**Fig. 2:**
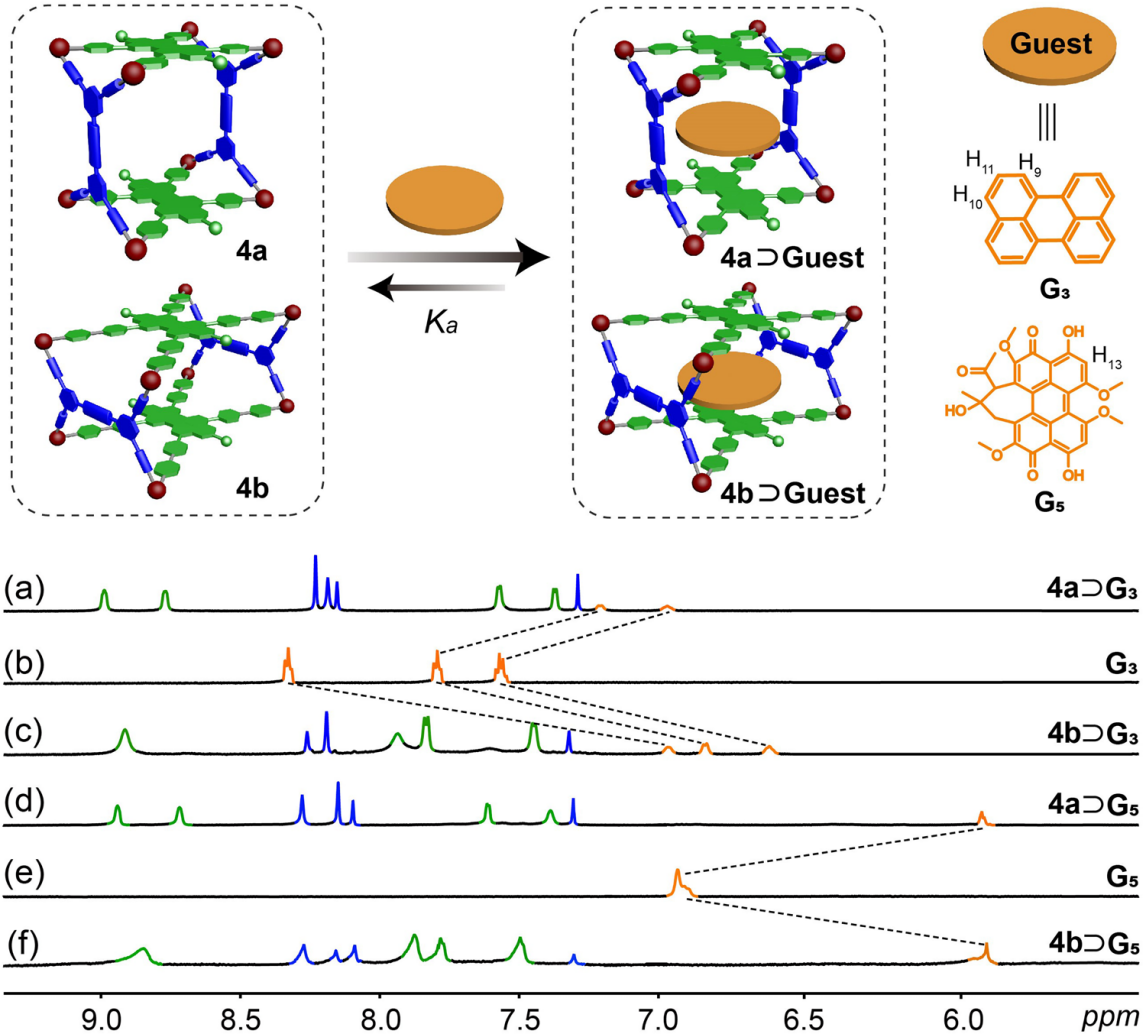
Cartoon illustrations of the complexation. Partial ^1^H NMR spectra (600 MHz, CD_3_CN, 295 K) of **a Cage 4a** ⊃ **G**_**3**_, **b G**_**3**_, **c Cage 4b** ⊃ **G**_**3**_, **d Cage 4a** ⊃ **G**_**5**_, **e G**_**5**_, **f Cage 4b** ⊃ **G**_**5.**_ [Host] = [Guest] = 2 mM

Considering that **Cage 4a** and **Cage 4b** possess electron-deficient PDI faces and available cavities, their complexation with polycyclic aromatic hydrocarbons (PAHs) including pyrene (**G**_**1**_), triphenylene (**G**_**2**_), perylene (**G**_**3**_), and coronene (**G**_**4**_) was first studied. The ^1^H NMR spectra (Figs. 2 and S10) revealed that all complexes underwent rapid exchange on the NMR time scale, which was because the open windows of the metallacages would allow guest molecules to readily enter and exit. The protons of all the guests experienced significant upfield chemical shifts, indicative of the effective host–guest complexation by **Cage 4a** and **Cage 4b**. Job’s plots (Figs. S11 − S18), based on UV/vis spectroscopic absorbance data, affirmed that the complexes of **Cage 4a** and **Cage 4b** with **G**_**1**_, **G**_**2**_, **G**_**3,**_ and **G**_**4**_ in solution maintained a 1:1 stoichiometry. Fluorescence titration experiments (Figs. S19 − S26) were conducted to determine the association constants (*K*_*a*_) of these complexes in solution. It was observed that *K*_*a*_ of **Cage 4b** was almost an order of magnitude larger than those of **Cage 4a** for all the four guests in acetonitrile (Table 1). This enhanced affinity can be attributed to the shorter distance between the two PDI faces of **Cage 4b**, which facilitates stronger π–π stacking interactions between the electron-deficient PDI and the electron-rich PAHs.

**Table 1:** Association constants between **Cage 4a**, **Cage 4b**, and different guests

	Association Constant (*K*_*a*_/M^−1^) CH_3_CN	
	Cage 4a	Cage 4b
G_1_	(2.09 ± 0.04) × 10^3^	(7.30 ± 0.15) × 10^4^
G_2_	(8.30 ± 0.18) × 10^2^	(7.29 ± 1.41) × 10^4^
G_3_	(5.25 ± 0.30) × 10^3^	(6.65 ± 0.34) × 10^4^
G_4_	(1.21 ± 0.03) × 10^4^	(6.69 ± 1.41) × 10^5^
G_5_	(8.91 ± 0.21) × 10^3^	(1.83 ± 0.03) × 10^4^
	^a^(5.40 ± 0.21) × 10^4^	^a^(1.51 ± 0.04) × 10^5^

^a^CH_3_CN/H_2_O (1:1) was used as the solvent

The above results encouraged us to explore the potential of the metallacages for encapsulating planar photosensitizer Hypocrellin A (**G**_**5**_). The complexation between **Cage 4a**/**Cage 4b** and **G**_**5**_ was initially investigated using ^1^H NMR spectroscopy (Fig. 2). Significant upfield shifts were observed for protons H_13_ of **G**_**5**_ owing to the shielding effect of the metallacages, indicating good host−guest complexation. Job’s plots (Figs. S27 and S28) and ESI mass spectra (Fig. S29) both supported the 1:1 binding stoichiometry of **Cage 4a** ⊃ **G**_**5**_ and **Cage 4b** ⊃ **G**_**5**_. For example, three peaks at m/z = 1232.9257, 1509.0952, and 1923.8585 were observed, which was attributed to [**Cage 4b** ⊃ **G**_**5**_ − 6OTf]^6+^, [**Cage 4b** ⊃ **G**_**5**_ − 5OTf]^5+^, and [**Cage 4b** ⊃ **G**_**5**_ − 4OTf]^4+^, respectively. The *K*_*a*_ of **Cage 4a** ⊃ **G**_**5**_ and **Cage 4b** ⊃ **G**_**5**_ in acetonitrile (Fig. S30 and S31) was determined to be (8.91 ± 0.21) × 10^3^ and (1.83 ± 0.03) × 10^4^ M^−1^, respectively. Notably, such values increased into (5.40 ± 0.21) × 10^4^ and (1.51 ± 0.04) × 10^5^ M^−1^, respectively, in water/acetonitrile (1:1), due to the solvophobic effect of **G**_**5**_ in aqueous environments [63, 64], which is highly advantageous for biological applications.

**Fig. 3:**
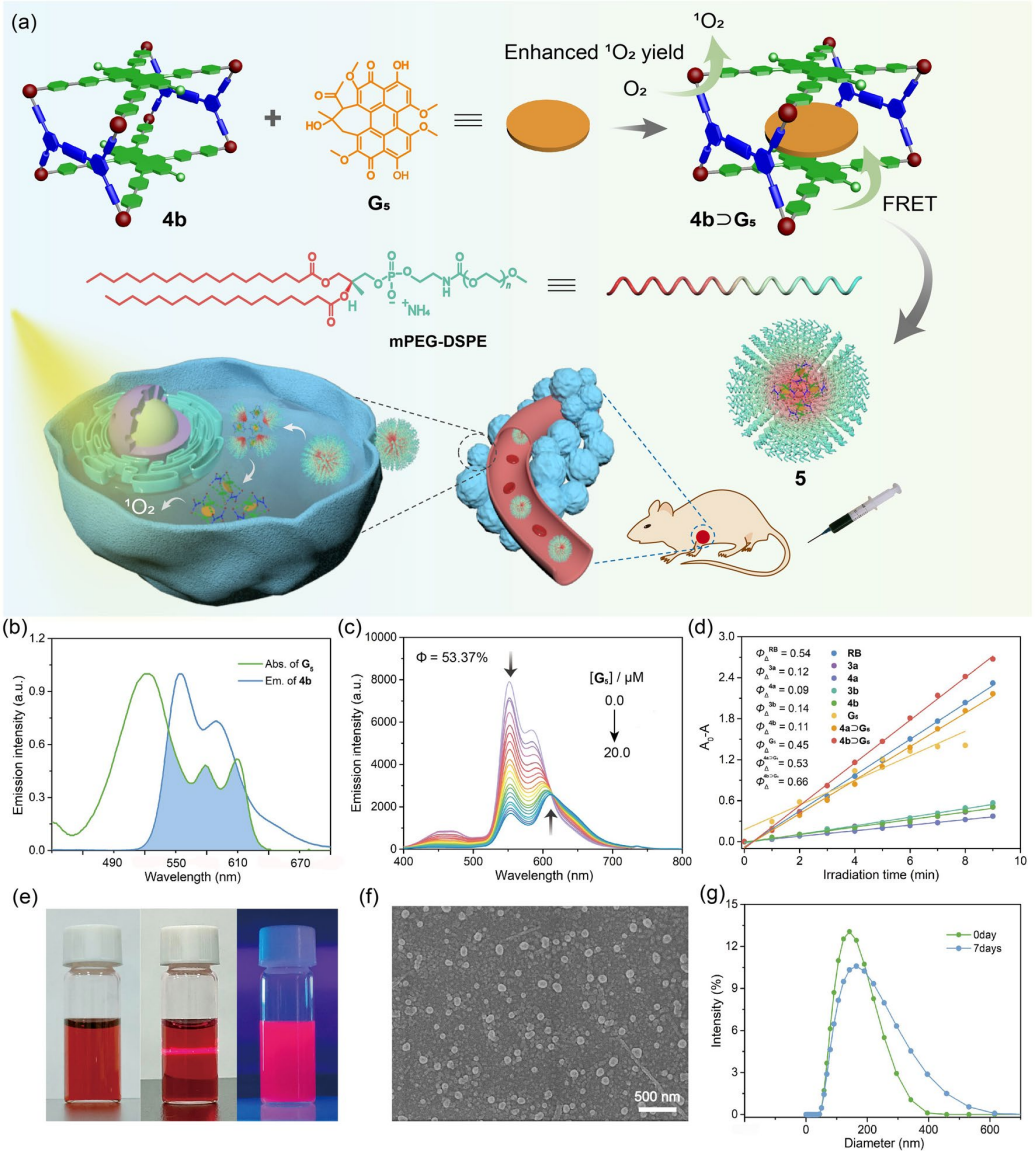
**a** Cartoon illustration of the cancer photochemotherapy of **NPs 5** self-assembled from **Cage 4b** ⊃ **G**_**5**_ and *m*PEG-DSPE2000. **b** Normalized absorption spectrum of **G**_**5**_ and emission spectrum of **Cage 4b**. **c** Fluorescence spectra of **Cage 4b** and **G**_**5**_ in a mixture of CH_3_CN and water (*v*/*v* = 1/1) with different concentrations of **G**_**5**_ (*λ*_ex_ = 365 nm, c = 10 µM for **Cage 4b**). **d** Plots of the absorption decays of DPBF at 410 nm versus the irradiation time in the presence of **3a**, **Cage 4a**, **3b**, **Cage 4b**, **G**_**5**_, **Cage 4a** ⊃ **G**_**5**_, or **Cage 4b** ⊃ **G**_**5**_ (*λ*_ex_ = 520 nm). **e** Photographs of **NPs 5,** Tyndall effect and **NPs 5** in CH_3_CN and water (*v*/*v* = 1/1) after illumination. **f** SEM image of **NPs 5**. **g** Particle size distributions of **NPs 5** measured by DLS (*c* = 1.0 mM)

Since the absorption of **G**_**5**_ exhibited a substantial overlap with the emission of **Cage 4a** and **Cage 4b** (Figs. 3b and S32a), FRET may occur from the metallacages to **G**_**5**_ [65, 66]. As the gradual addition of **G**_**5**_ to the solution of **Cage 4a** or **Cage 4b**, the emission of the metallacages decreased while that of the **G**_**5**_ increased (Figs. 3c and S32b), providing compelling evidence for the efficient energy transfer between **G**_**5**_ and **Cage 4b**. The formation of host–guest complexes shortened the distance between the metallacage donors and** G**_**5**_, offering good energy transfer efficiency (Φ_ET_). Φ_ET_ of **Cage 4a** ⊃ **G**_**5**_ and **Cage 4b** ⊃ **G**_**5**_ were calculated to be 36.99% and 53.37%, respectively. The higher Φ_ET_ of **Cage 4b** ⊃ **G**_**5**_ compared with that of **Cage 4a** ⊃ **G**_**5**_ is attributed to the higher binding affinity of **Cage 4b** ⊃ **G**_**5**_, because higher binding affinity would give higher complexation ratio for the complexes and thus increase the energy transfer efficiency. Such FRET process is expected to boost the light-harvesting performance and enhance the generation of ^1^O_2_.

To validate the hypothesis, electron spin resonance (ESR) spectroscopy was utilized to investigate the generation of ^1^O_2_ through sensitization of **Cage 4a ⊃ G**_**5**_ and **Cage 4b** ⊃ **G**_**5**_ using 2,2,6,6-tetramethylpiperidine (TEMP) as a ^1^O_2_ sensor, which was capable of generating stable tetramethylpiperidine oxide radical (TEMPO) upon trapping ^1^O_2_. All complexes exhibited a 1:1:1 triplet signal upon light irradiation (Fig. S33), which was consistent with the ^1^O_2_ signal generated by the complexes. Compared to **Cage 4b** ⊃ **G**_**5**_, the signal from **Cage 4a** ⊃ **G**_**5**_ appeared relatively weak. In addition, the ^1^O_2_ generation quantum yields of **3a**, **Cage 4a**, **3b**, **Cage 4b**, **G**_**5**_, **Cage 4a** ⊃ **G**_**5**_, and **Cage 4b** ⊃ **G**_**5**_ were measured using a reactive ^1^O_2_ scavenger, 1,3-diphenylisobenzofuran (DPBF) [67]. Time-dependent UV/vis absorption spectra of a mixture solution of DPBF and different species upon irradiation at 520 nm were recorded (Fig. S34). A gradual decrease in the characteristic absorption band centered at 410 nm for DPBF was observed with increasing exposure time, indicating the accumulation of ^1^O_2_. The absorption bands corresponding to **Cage 4a**, **Cage 4b**, **Cage 4a** ⊃ **G**_**5**_, and **Cage 4b** ⊃ **G**_**5**_ remained almost unchanged, suggesting their good photostability. The ^1^O_2_ quantum yields (Φ_Δ_) of all the compounds were calculated using Rose Bengal (RB) with a known efficiency (Φ_ΔRB_ = 0.54) as the reference. The Φ_Δ_ values were determined to be 12%, 9%, 14%, 11%, 45%, 53%, and 66% for **3a**, **Cage 4a**, **3b**, **Cage 4b**, **G**_**5**_, **Cage 4a** ⊃ **G**_**5,**_ and **Cage 4b** ⊃ **G**_**5**_, respectively (Fig. 3d). Based on these results, both **Cage 4a**/**Cage 4b** and **G**_**5**_ exhibited the capacity for ^1^O_2_ generation. Additionally, the FRET between metallacages and **G**_**5**_ further enhanced the efficiency of ^1^O_2_ generation for complexes **Cage 4a** ⊃ **G**_**5**_ and **Cage 4b** ⊃ **G**_**5**_. Notably, **Cage 4b** ⊃ **G**_**5**_ demonstrated a higher Φ_Δ_ compared with **Cage 4a** ⊃ **G**_**5**_, attributed to its better host−guest complexation.

Based on the outstanding ^1^O_2_ generation capability of **Cage 4b** ⊃ **G**_**5**_, its application for cancer photodynamic therapy was further explored. 1,2-Distearoyl-phosphatidylethanolamine (DSPE)/polyethylene glycol (PEG) conjugate (*m*PEG-DSPE2000) [68], a commonly used polymer, was assembled with complex **Cage 4b** ⊃ **G**_**5**_ to form nanoparticles **NPs 5**, which were further used for further biological experiments (Fig. 3a). The photo image (Fig. 3e) of **NPs 5** presented obvious “Tyndall effect” in solution, which indicated the successful preparation of dispersed colloidal nanoparticles. The size and morphology of **NPs 5** were examined using scanning electron microscopy (SEM) and dynamic light scattering (DLS), respectively. The SEM image (Fig. 3f) revealed that **NPs 5** exhibited micellar structures with diameters of 140–150 nm. DLS analysis (Fig. 3g) indicated an average hydrodynamic diameter of 142 nm, which agreed well with the SEM results. It is believed that nanoparticles within this size range can exhibit an enhanced permeability and retention effect [69], potentially augmenting their uptake and retention within tumors and increasing their anticancer activity. The size distribution of nanoparticles appeared broad, suggesting that these amphiphilic structures may readily aggregate in aqueous solution. No notable alterations in size (Fig. 3g) or absorption (Fig. S35) were observed for these nanoparticles even after 7 days, demonstrating the exceptional stability of complex-loaded **NPs 5**, attributed to the protective role of the amphiphilic *m*PEG-DSPE, which securely houses the complex within the hydrophobic interior.

### Cell Imaging and Anticancer Study

**Fig. 4:**
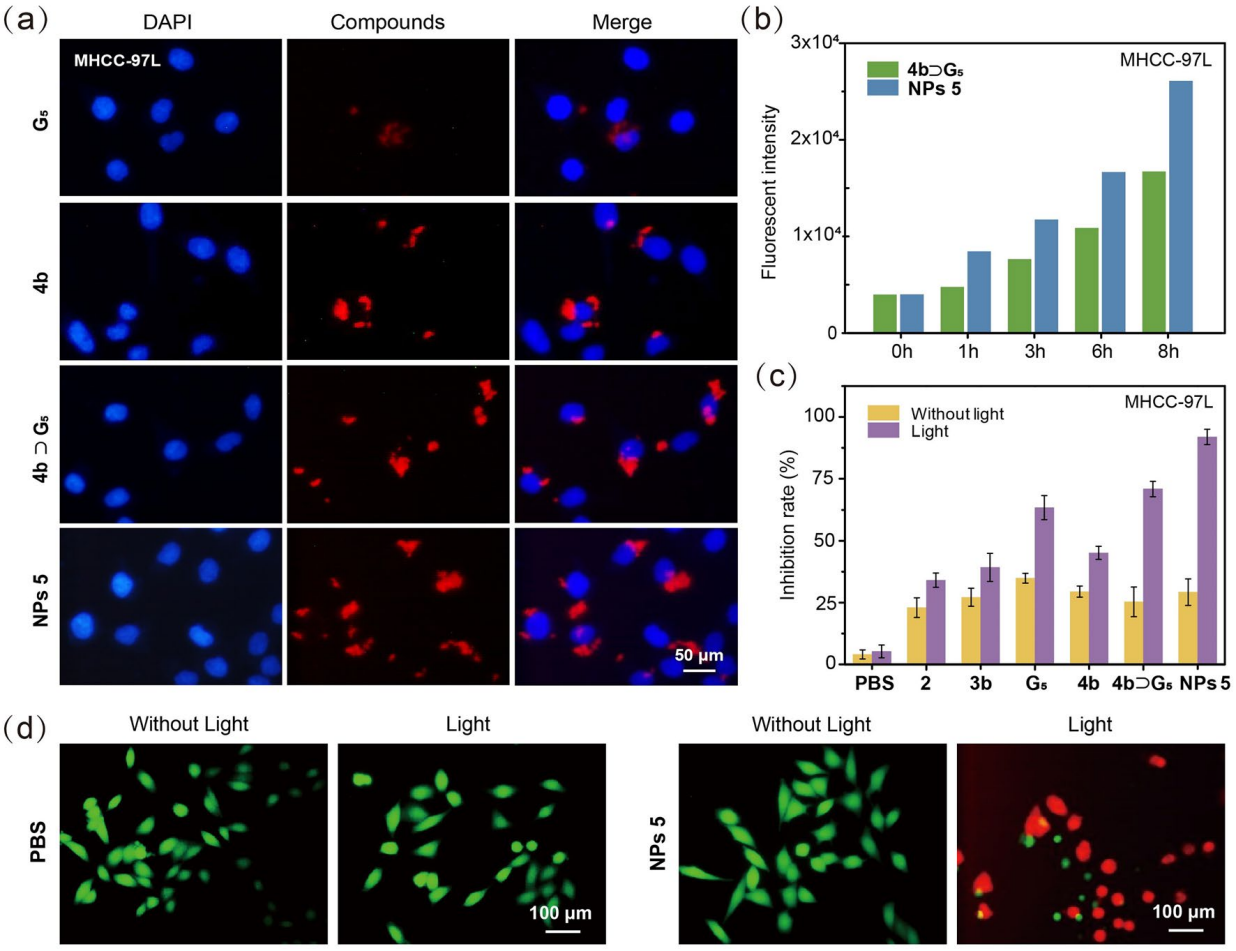
**a** CLSM images of MHCC-97L cells after the incubation with DAPI and **G**_**5**_, **Cage 4b**, **Cage 4b** ⊃ **G**_**5,**_ or **NPs 5**. **b** Fluorescence intensity of cells incubated with **4b** ⊃ **G**_**5**_ or **NPs 5** at different times. **c** Cell inhibition of MHCC-97L cells incubated with **3b**, **G**_**5**_, **Cage 4b**, **Cage 4b** ⊃ **G**_**5**_, or **NPs 5** without/with light irradiation. **d** CLSM images of MHCC-97L cells after the incubation with PBS and **NPs 5** without/with light irradiation (excitation wavelength: FDA 488 nm, PI 543 nm; emission filter: FDA 500–550 nm, PI 550–650 nm)

The UV/vis absorption and emission spectra of **3a**, **Cage 4a**, **3b**, **Cage 4b**, **G**_**5**_, **Cage 4a** ⊃ **G**_**5**_, and **Cage 4b** ⊃ **G**_**5**_ were recorded to assess their optical characteristics (Fig. S36). Ligand **3a** exhibited three absorption bands centered at 466, 496, and 534 nm, which was consistent with the typical absorption of PDI derivatives [70, 71]. The corresponding molar absorption coefficients (*ε*) were determined to be 1.50 × 10^4^, 3.21 × 10^4^, and 4.08 × 10^4^ M^−1^ cm^−1^, respectively. Likewise, ligand **3b**, **Cage 4a,** and **Cage 4b**, complexes **Cage 4a** ⊃ **G**_**5**_ and **Cage 4b** ⊃ **G**_**5**_ all displayed similar absorption bands in the visible region. Furthermore, two distinct emission peaks were observed for **3a** at wavelengths of 515 and 551 nm. In contrast, **3b** exhibited much weaker emission with peaks centered at 572 and 621 nm, which is probably due to the increased molecular motions in **3b** compared with **3a**. **Cage 4a** and **Cage 4b** exhibited two emission bands centered at 516 and 560 nm, and 556 and 597 nm, respectively. The fluorescence quantum yields (Φ_F,_ Figs. S37–S41) of **3a**, **3b**, **Cage 4a**, **Cage 4b**, and **G**_**5**_ were determined to be 93.91%, 0.96%, 34.06%, 4.93%, and 3.68%, respectively. As for **Cage 4a** ⊃ **G**_**5**_ and **Cage 4b** ⊃ **G**_**5**_, the complexation leads to bathochromic shift and increased emission via FRET, with the Φ_F_ values of 42.09% and 27.19%, respectively (Figs. S42 and S43). Therefore, **Cage 4b**, **Cage 4b** ⊃ **G**_**5**_, and **5** were further used as contrast agents for bioimaging. Liver cancer MHCC-97L cells and lung cancer NCI-H460 cells were stained with DAPI and treated with above-mentioned compounds simultaneously. After 6 h, the intensity of the fluorescence reached the maximum value which did not increase as time went by, so the images were taken at 6-h post incubation (Figs. 4a and S44) using confocal laser scanning microscopy (CLSM). Based on the merged figures, bright red fluorescence originating from these compounds was observed within the cells. The cellular uptake of complex **Cage 4b** ⊃ **G**_**5**_ and **NPs 5** was assessed through flow cytometry (FCM) analysis. In both MHCC-97L and NCI-H460 cells (Figs. 4b and S45 − S47), stronger emission was observed for **NPs 5,** suggesting that the cellular uptake of **NPs 5** was better than complex **Cage 4b** ⊃ **G**_**5**_ after incubation. Collectively, these findings confirm the suitability of **Cage 4b**, **Cage 4b** ⊃ **G**_**5**_, and **NPs 5** as contrast agents for cell imaging.

Considering the ^1^O_2_ generation capacity and good stability of **G**_**5**_ and PDI derivatives, **3b**, **G**_**5**_, **Cage 4b**, **Cage 4b** ⊃ **G**_**5**_, and **NPs 5** were employed to study the intracellular ^1^O_2_ production. Flow cytometry (FCM) experiments (Figs. S48 and S49) utilizing 2,7-dichlorodi-hydrofluorescein diacetate (DCFH-DA) as a reactive ^1^O_2_ scavenger [72] revealed that only cells treated with light exhibited good fluorescence, indicating that all the compounds were capable of producing ^1^O_2_ under light irradiation. Significantly, the fluorescence intensity of **Cage 4b** ⊃ **G**_**5**_ and **NPs 5** was much stronger than that of other compounds, suggesting that these two compounds possessed the highest ^1^O_2_ generation capacity among all tested compounds in cells, consistent with their ^1^O_2_ generation quantum yields in solution (Fig. 3d). Moreover, due to the higher cellular uptake of **NPs 5** compared to **Cage 4b** ⊃ **G**_**5**_, **NPs 5** exhibited better intracellular ^1^O_2_ production.

The anticancer efficacy for PDT involving **2**, **3b**, **G**_**5**_, **Cage 4b**, **Cage 4b** ⊃ **G**_**5**_, and **NPs 5** against two human cancer cell lines (MHCC-97L and NCI-H460 cells) was assessed using 3-(4’,5’-dimethylthiazol-2’-yl)-2,5-diphenyltetrazolium bromide (MTT) assay (Figs. 4c and S50). In order to ensure the comparability of test results, the absolute concentration of all the compounds was set to be 1.25 μM. Compared with compounds without light treatment, all tested substances exhibited significantly enhanced anticancer activities upon light irradiation. Taking **NPs 5** as an example, in the absence of irradiation, a small amount of apoptotic and necrotic cells was detected, which may be related to the chemotherapeutic effect of the platinum(II) contained in the metallacage. However, when treated with laser irradiation (50 mW cm^−2^, white light, 1 min), the inhibition rate increased from 29 to 92% for MHCC-97L cells. For NCI-H460 cells, **NPs 5** achieved 92% inhibition rate under irradiation. Notably, **NPs 5** showed the highest cytotoxicity among these compounds. This is likely attributed to the occurrence of FRET from **Cage 4b** to **G**_**5**_, leading to an increase in the efficiency of ^1^O_2_ generation in **Cage 4b** ⊃ **G**_**5**_. The photodynamic therapeutic effect of **NPs 5** was further demonstrated by staining live cells and apoptotic cells with fluorescein diacetate (FDA) and propidium iodide (PI), respectively. As depicted in Fig. 4d, the control groups (PBS, PBS with light irradiation, and **NPs 5** without light) exhibited green fluorescent live cells. Only the experimental group (**NPs 5** with laser irradiation) predominantly showed red fluorescence, indicating apoptotic cells, providing further evidence of effective photodynamic therapy.

**Fig. 5:**
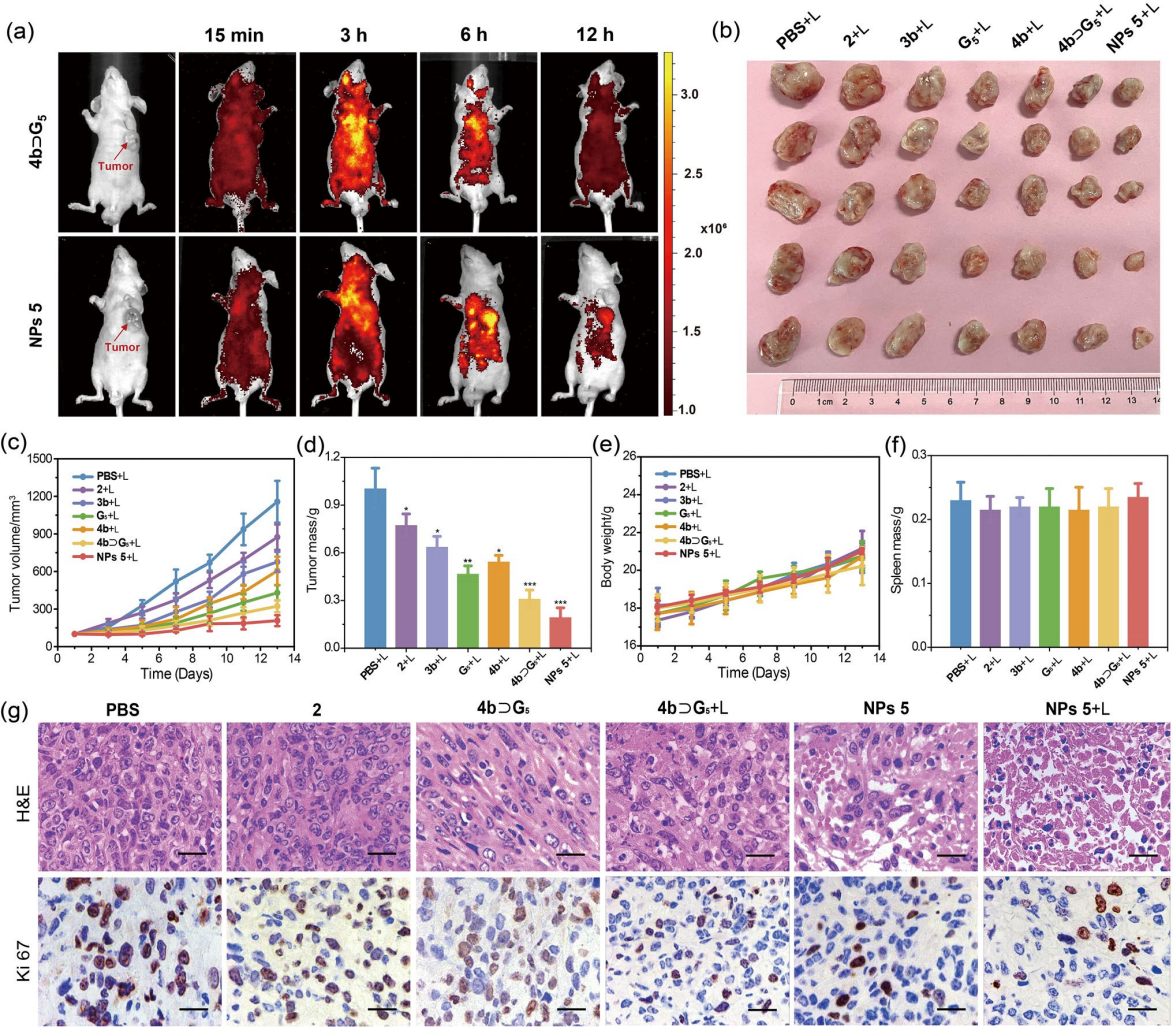
**a** In vivo fluorescence imaging of the tumor-bearing mice after tail vein injection of **Cage 4b** ⊃ **G**_**5**_ and **NPs 5**. **b** Digital photos of the final tumor tissues harvested from the mice treated with different formulations at day 14. **c** Tumor volume changes, **d** tumor mass (∗*p* < 0.1, ∗  ∗*p* < 0.01, ∗  ∗  ∗*p* < 0.001), **e** body weight changes, and **f** spleen mass after injection of various formulas with 520 nm (50 mW cm^−2^) irradiation (35 tumor-bearing female nude mice were divided into seven groups, *n* = 5/group). **g** H&E and Ki 67 staining of postoperative sections collected from different groups treated tumor tissues after 14 d therapies (The scale bar is 100 μm)

In vivo experiments were further conducted to evaluate the theranostic activities of **2**, **3b**, **G**_**5**_, **Cage 4b**, **Cage 4b** ⊃ **G**_**5**_, and **NPs 5**. In this study, MHCC-97L tumor-bearing nude mice with subcutaneous xenograft tumor models were employed. Initially, in vivo fluorescence imaging of mice was carried out after tail vein injection of these compounds to evaluate their uptake and biodistribution capability (Figs. S51 and S52). A noticeable fluorescence signal was concentrated at the tumor site in mice after the treatment with **3b**, **G**_**5**_, **Cage 4b**, **Cage 4b** ⊃ **G**_**5**_, or **NPs 5**. In contrast, negligible fluorescence signal was detected in other organs such as the liver and kidneys, indicating a significant accumulation of compounds in the tumor area 6-h post injection. Notably, the fluorescence signal of **NPs 5** was notably higher than that of other compounds, suggesting that these nanoparticles exhibited long-term fluorescence and possessed excellent tumor accumulation and retention capabilities. The strong fluorescence exhibited by **NPs 5** facilitated imaging techniques for monitoring the pharmacokinetics in mice with tumors. Following injection, **NPs 5** displayed sustained systemic distribution, and the fluorescence of **NPs 5** localized in tumor tissues progressively increased over time (Fig. 5a). Fluorescence imaging conducted at 6-h post injection revealed heightened fluorescence at the tumor site compared to normal organs. This signal remained for at least 12-h post injection, indicating obvious biodistribution of **NPs 5** within the sample. Conversely, mice administered with **Cage 4b ⊃ G**_**5**_ displayed a more rapid decline in fluorescence signal throughout the body. This can be attributed to the prolonged circulation times of PEGylated **NPs 5**, which markedly enhanced their permeability and retention within tumors.

The in vivo antitumor efficacy of the photochemotherapy was further assessed. The mice were randomly divided into seven groups (*n* = 5/group) when the tumor volume reached ∼100 mm^3^ and were subcutaneously injected with PBS buffer, **2**, **3b**, **G**_**5**_, **Cage 4b**, **Cage 4b** ⊃ **G**_**5**_, or **NPs 5**, all at a dose of 2 mg kg^−1^ in platinum weight, then treated with laser irradiation (50 mW cm^−2^ for 30 s). The average tumor size of the different groups was observed every 2 days for 2 weeks to evaluate the therapeutic effect. All the test groups, including **3b**, **G**_**5**_, **Cage 4b**, **Cage 4b** ⊃ **G**_**5,**_ and **NPs 5**, exhibited better antitumor activities compared to the control group administered with PBS and **2**, as evidenced by the decreased size of the tumor after treatment (Fig. 5b). As depicted in Fig. 5c, weak therapeutic effects were observed for the mice that only underwent single chemotherapy (**2**) or single PDT (**3b** and **G**_**5**_) compared to the PBS group. An exceptional antitumor outcome was achieved for the photochemotherapy group (**Cage 4b** ⊃ **G**_**5**_ and **NPs 5**). Notably, the mice treated with **NPs 5** exhibited the highest tumor growth inhibition at 14-day post treatment, confirmed by the smallest tumor volume among all the test groups, which aligned with the tumor mass outcome (Fig. 5d). Moreover, the body weights of the mice remained very similar (Fig. 5e). There was no significant reduction in body weight, and no distinct signs of toxic effects such as changes in urination or nervous behavior were observed for all the tested compounds. The spleen mass (Fig. 5f) remained nearly unchanged, indicating that these compounds can be applied as therapeutic agents for cancer treatment.

Hematoxylin and eosin (H&E) staining assay was also conducted to assess the proliferation and apoptosis of cells in the tumor tissue (Fig. 5g). All treatment groups exhibited varying degrees of necrosis compared to the PBS group, indicating that all tested compounds possessed certain antitumor activities. Remarkably, **NPs 5** with laser irradiation resulted in noticeable shrinkage and alterations in the tumor cells and the highest level of tumor apoptosis and necrosis, signifying a pronounced inhibitory effect on tumor proliferation. Furthermore, Ki67-positive immunohistochemical staining was utilized, where areas of cell proliferation were marked by brown spots in the captured images in Fig. 5g. Notably, the group treated with **NPs 5** under laser irradiation showed the most substantial decrease in the count of Ki67-positive tumor cells compared with other treatment groups. In essence, these results unequivocally confirmed the amplified synergistic therapeutic performance achieved through the combination of chemotherapy and laser irradiation-activated PDT. These results suggested that the host–guest complexation between the metallacages and photosensitizers, which has been demonstrated to increase the ^1^O_2_ generation via FRET, played a pivotal role to increase the efficacy of cancer photochemotherapy. The routine blood test analysis and blood biochemical assay were conducted to further assess the potential long-term toxicity of **NPs 5** in vivo. All markers remained within normal ranges (Figs. S53 and S54), indicating no significant toxicity or inflammatory response. These findings emphasize the efficacy of combining chemotherapy and PDT in tumor treatment, thereby improving the survival quality of mice and extending their lifespan.

## Conclusions

In summary, two emissive perylene diimide metallacages with different cavity sizes were prepared and further utilized for complexing hypocrellin-type photosensitizer through host–guest interactions. The aggregation of hypocrellin A in an aqueous solution was suppressed after complexation with the metallacage. Noticeably, the efficient host–guest complexation shortened the distance between the metallacages and hypocrellin A, offering effective FRET from the metallacages to hypocrellin A and increasing the ^1^O_2_ generation quantum yields. Thus, the host–guest complexes were further assembled into supramolecular nanoparticles, demonstrating superior photodynamic activities for cancer therapy compared with sole metallacages and photosensitizer, as indicated by both in vitro and in vivo studies. This study offers an efficient strategy to address the photosensitivity limitation of conventional photosensitizers through the host–guest complexation-based FRET, which will promote the development of metallacage-based delivery system for cancer therapy.

## Supplementary Information

Below is the link to the electronic supplementary material.

Supplementary file1 (PDF 8185 KB)
