# Supplementary material for: Enhancing the Photosensitivity of Hypocrellin A by Perylene Diimide Metallacage-Based Host–Guest Complexation for Photodynamic Therapy
Source: Nanomicro Lett. 2024 Jun 25;16:226. doi: 10.1007/s40820-024-01438-w (PMC11199435; doi:10.1007/s40820-024-01438-w)
Supplement: Supplementary file 1 — Supplementary file1 (PDF 8185 KB) [file 40820_2024_1438_MOESM1_ESM.pdf]

Supporting Information for

Enhancing the Photosensitivity of Hypocrellin A by Perylene Diimide Metallacage-Based Host-Guest Complexation for Photodynamic Therapy

Rongrong Li [1,2,‡], Tianfeng Yang [2,‡], Xiuhong Peng [2,‡], Qian Feng[1], Yali Hou[1,3], Jiao Zhu[4], Dake Chu[4], Xianglong Duan [3,*], Yanming Zhang, [2,*] and Mingming Zhang[1,*]

[1]State Key Laboratory for Mechanical Behavior of Materials, Shaanxi International Research Center for Soft Matter, School of Materials Science and Engineering, Xi'an Jiaotong University, Xi'an 710049, P. R. China

[2]School of Pharmacy, Health Science Center, Xi'an Jiaotong University, Xi'an 710061, P. R. China

[3]Department of Rehabilitation Medicine, Shaanxi Provincial People's Hospital, Xi'an, Shaanxi, 710068, P. R. China

[4]Department of Gastroenterology, The First Affiliated Hospital of Xi'an Jiaotong University, Xi'an 710061, P. R. China

[‡]Rongrong Li, Tianfeng Yang and Xiuhong Peng contributed equally to this work.

*Corresponding authors. E-mail: mingming.zhang@xjtu.edu.cn (Mingming Zhang) ; zhang2008@xjtu.edu.cn (Yanming Zhang) ; duanxianglong@nwpu.edu.cn (Xianglong Duan)

S1 Materials and Methods

All reagents and deuterated solvents were used as purchased without further purification. Compounds **2**[S1], **7**[S2], **8**[S2] and **3a**[S3] were prepared according to the literature procedures. NMR spectra were recorded on a Bruker Avance 400 or 600 MHz spectrometer. ^{1}H NMR chemical shifts were recorded relative to residual solvent signals. ^{31}P{^{1}H} NMR chemical shifts were referenced to an external unlocked sample of 85% H_3PO_4 (δ 0.0). Mass spectra were recorded on a Micromass Quattro II triple-quadrupole mass spectrometer using electrospray ionization with a MassLynx operating system. The UV-vis experiments were conducted on a Lambd 950 absorption spectrophotometer. The fluorescent experiments were conducted on an Edinburgh FLS9 or a Hitachi F-7000 fluorescence spectrophotometer. X-ray diffraction analysis was conducted on a Bruker SMART APEX II diffractometer at 108 K with graphite-monochromated Mo Kα radiation (λ = 0.71073 Å) or a Bruker D8 VENTURE PHOTON III MetalJet, in which crystals were frozen in paratone oil inside a cryoloop under a cold stream of N_2. An empirical absorption correction using SADABS was applied for all data. The structures were solved and refined to convergence on F2 for all independent reflections by the

full-matrix least squares method using the OLEX2 1.2. Confocal laser scanning microscopy (CLSM) was performed with a Leica TCS SP8 STED 3X Super-resolution Confocal Microscope. Fluorescence titration experiments were performed by the addition of different amounts of guests into the $CH_3CN/CHCl_3$ (1:1) or CH_3CN solution of metallacages and the concentration of the metallacages was fixed to be 10.0 μM. The data was fitted according to the following equation:

$$\delta = (\Delta\delta_{max}/[H]_0)\ (0.5[G] + 0.5\ ([H]_0+1/K_a) - (0.5([G]^2+(2[G](1/K_a-[H]_0)) + (1/K_a+[H]_0)^2)^{0.5}))$$

Where δ is the fluorescence changes of metallacages upon the addition of guests, $\Delta\delta_{max}$ is the maximum fluorescence intensity change when the metallacages are totally complexed, $[H]_0$ is the fixed concentration of metallacages (10.0 μM), [G] is the concentration of the guests. All of these fluorescence titration experiments have been repeated three times.

S2 Synthetic Procedures and Characterization Data

S2.1 Synthesis of ligand 3b

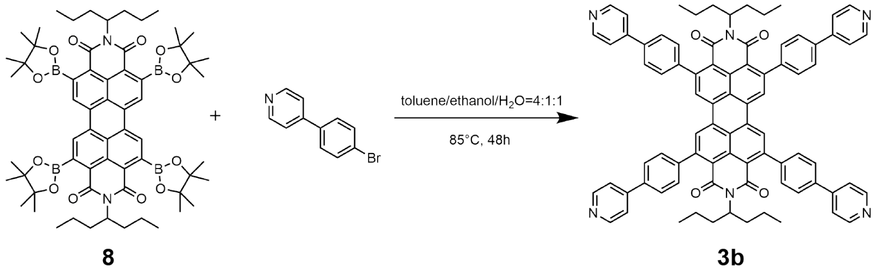

To compound **8** (1.00 g, 0.92 mmol), 4-(4-Bromophenyl)pyridine (1.72 g, 7.34 mmol), anhydrous Cs_2CO_3 (2.39 g, 7.34 mmol) and $Pd(PPh_3)_4$ (0.21 g, 0.18 mmol) in toluene (60.0 mL), H_2O (12.0 mL) and ethanol (12.0 mL) were added. Then the reaction mixture was stirred at 85 °C for 48 h under nitrogen atmosphere. After being cooled to room temperature, the product was concentrated to give a crude product which was purified by flash column chromatography with CH_2Cl_2:CH_3OH (50:1, *v*/*v*) as the eluent to afford compound **3b** (0.88 g, 80%) as an orange powder. ^{1}H NMR (600 MHz, $CDCl_3$) δ 8.71 (d, *J* = 4.5 Hz, 8H), 8.45 (s, 4H), 7.81 (d, *J* = 8.0 Hz, 8H), 7.61 (d, *J* = 5.5 Hz, 8H), 7.59 (d, *J* = 8.0 Hz, 8H), 5.00 (s, 2H), 2.10 (dd, *J* = 13.6, 7.7 Hz, 4H), 1.66 – 1.63 (m, 5H), 1.31 (dd, *J* = 15.1, 7.6 Hz, 9H), 0.89 (t, *J* = 7.3 Hz, 12H). ^{13}C NMR (243 MHz, $CDCl_3$, 295 K) 150.38, 147.67, 137.69, 132.73, 131.19, 128.90, 127.18, 121.64, 54.41, 34.13, 31.52, 31.45, 29.71, 20.07, 14.09. ESI-HR-MS: *m/z* 1199.5179 [**3b** + H]$^+$, calcd. for $[C_{82}H_{66}N_6O_4]^+$, 1199.5212.

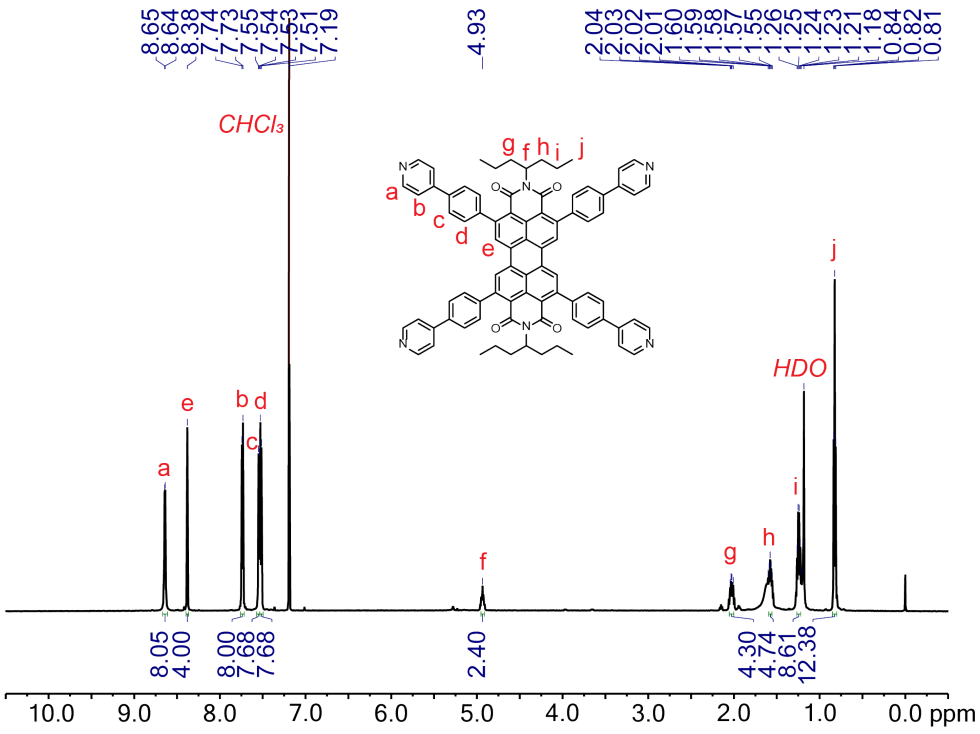

Fig. S1 ^{1}H NMR spectrum (600 MHz, $CDCl_3$, 295 K) recorded for **3b**

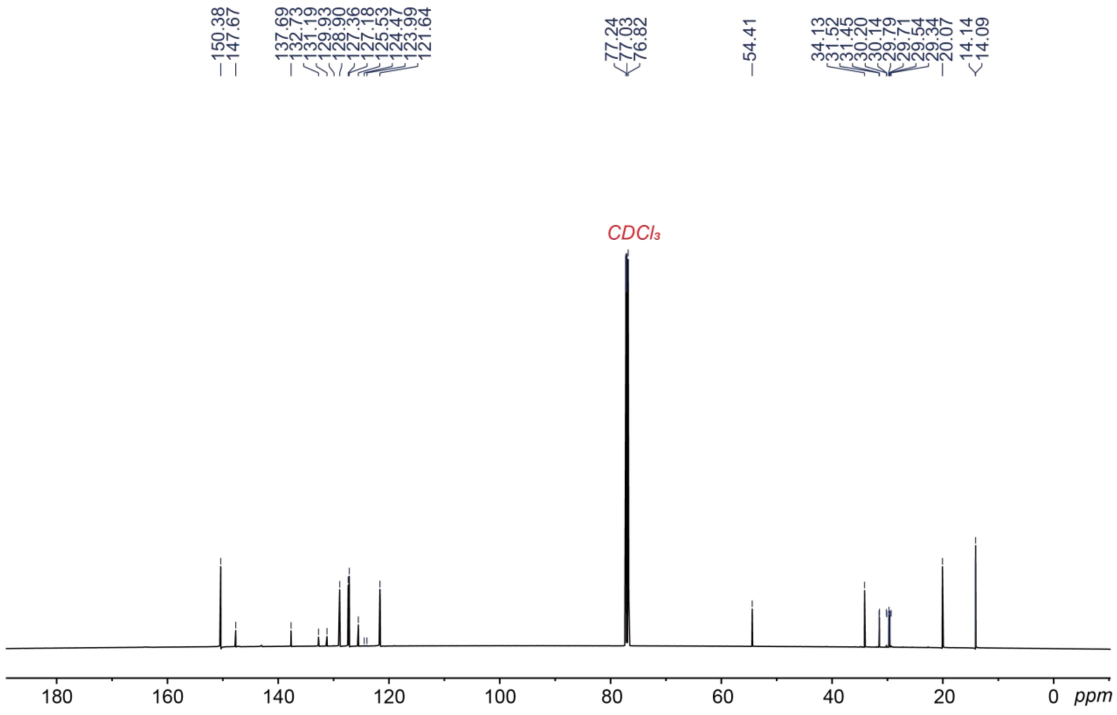

Fig. S2 ^{13}C NMR spectrum (151 MHz, $CDCl_3$, 295 K) recorded for **3b**

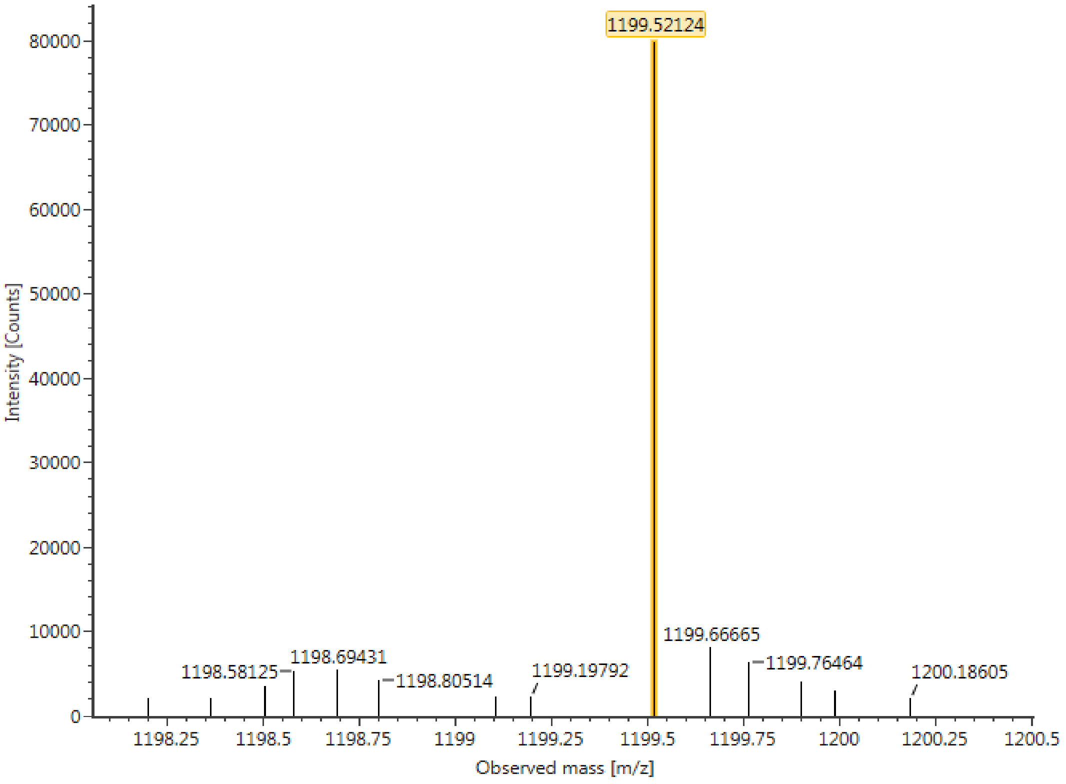

Fig. S3 ESI-HR-MS spectrum of **3b**

S2.2 Synthesis of metallacage 4a

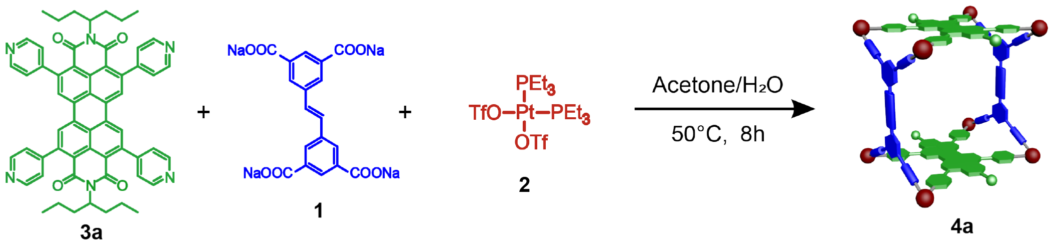

Fig. S4 Self-assembly of **Cage 4a**

3a (3.00 mg, 3.35 μmol), tetracarboxylate ligand **1** (1.49 mg, 3.35 μmol), and cis-$(PEt_3)_2Pt(OTf)_2$ (9.78 mg, 13.41 μmol) were mixed in a 1:1:4 molar ratio and dissolved in acetone/water (6.0 mL, 4:1, *v*/*v*). The whole reaction mixture was heated at 50°C for 8 h and then cooled to room temperature. The solvent was removed by nitrogen flow. The residue was redissolved in CH_3CN (1.0 mL) and filtered, and the filtrate was poured into ethyl ether (10.0 mL) to give a precipitate, which was collected by centrifugation to give **Cage 4a** (15.00 mg, 93%) as an orange powder. ^{1}H NMR (600 MHz, CD_3CN) δ 9.01 (s, 8H), 8.76 (s, 8H), 8.38 (s, 8H), 8.18 (d, J = 34.2 Hz, 11H), 7.63 (s, 8H), 7.38 (s, 8H), 7.27 (s, 4H), 4.78 (s, 4H), 1.05 – 0.64 (m, 24H). ^{31}P{^{1}H}NMR (243 MHz, CD_3CN, 295 K): 5.36 ppm (d, $^2J_{P\text{-}P}$ =21.9 Hz, ^{195}Pt satellites, $^1J_{Pt\text{-}P}$ = 1660.3 Hz), -0.12 pp m (d, $^2J_{P\text{-}P}$ = 21.9 Hz, ^{195}Pt satellites, $^1J_{Pt\text{-}P}$ = 1660.3

Hz). ESI-TOF-MS: *m*/*z* 742.9245 [**Cage 4a** – 8OTf]$^{8+}$, 1040.5464 [**Cage 4a** − 6OTf]$^{6+}$, 1278.2423 [**Cage 4a** − 5OTf]$^{5+}$ and 1635.2900 [**Cage 4a** − 4OTf]$^{4+}$.

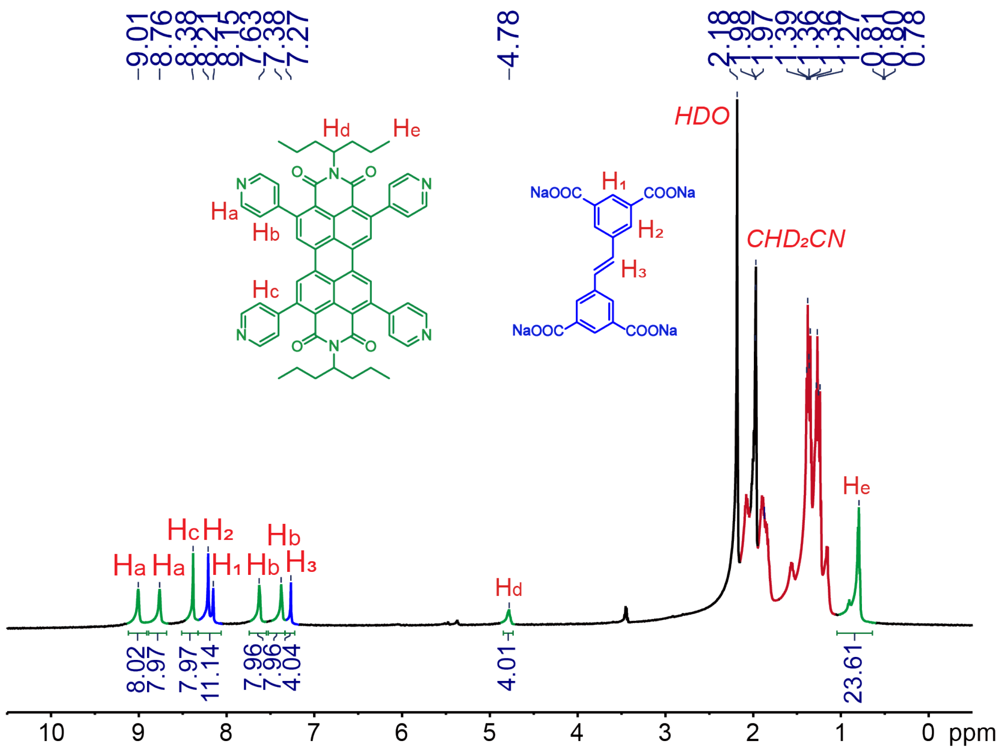

Fig. S5 ^{1}H NMR spectrum (600 MHz, $CDCl_3$, 295 K) recorded for **Cage 4a**

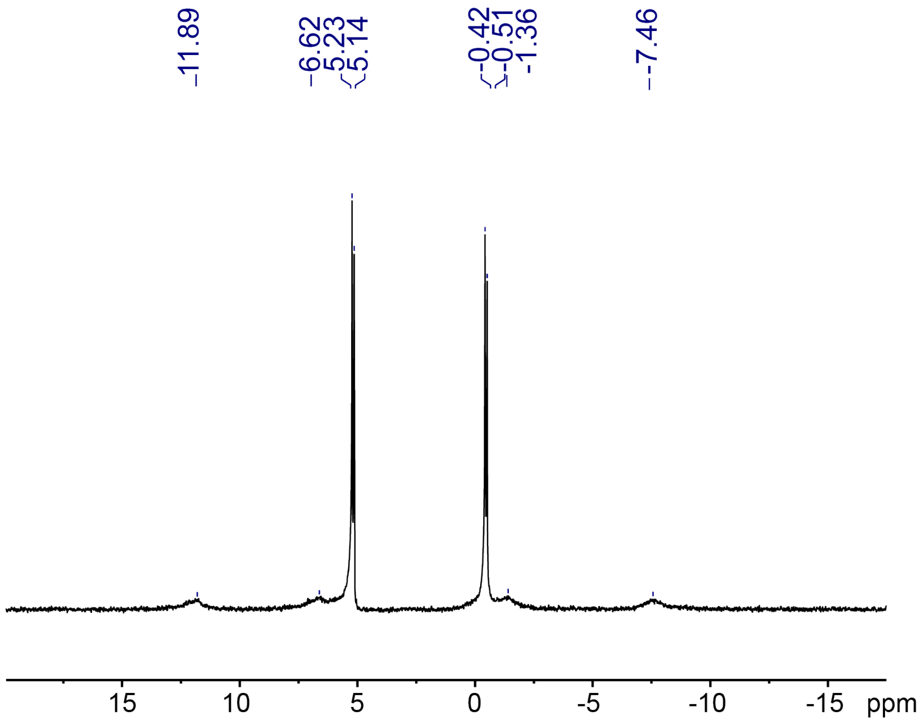

Fig. S6 $^{31}P\{^{1}H\}$ NMR spectrum (243 MHz, CD_3CN, 295 K) recorded for **Cage 4a**

S2.3 Synthesis of Cage 4b

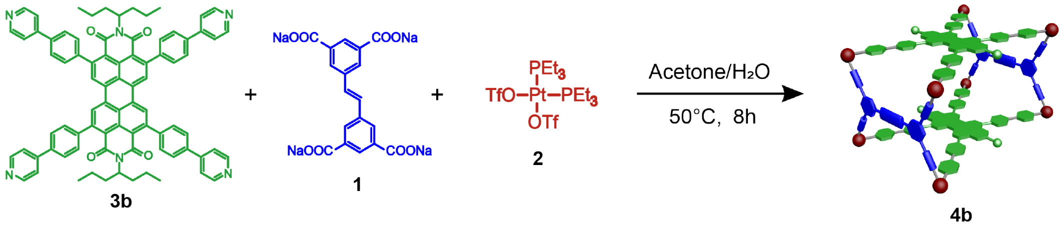

Fig. S7 Self-assembly of **Cage 4b**

3b (3.00 mg, 2.50 μmol), tetracarboxylate ligand **1** (1.11 mg, 2.50 μmol), and cis-$(PEt_3)_2Pt(OTf)_2$ (7.30 mg, 10.00 μmol) were mixed in a 1:1:4 molar ratio and dissolved in acetone/water (6.0 mL, 4:1, *v*/*v*). The whole reaction mixture was heated at 50°C for 8 h and then cooled to room temperature. The solvent was removed by nitrogen flow. The residue was redissolved in CH_3CN (1.0 mL) and filtered, and the filtrate was poured into ethyl ether (10.0 mL) to give a precipitate, which was collected by centrifugation to give **Cage 4b** (15.00 mg, 93%) as an orange powder. ^{1}H NMR (600 MHz, CD_3CN) δ 8.82 (s, 16H), 8.28 (s, 8H), 8.15 (s, 4H), 8.07 (s, 8H), 7.83 (d, J = 3.5 Hz, 16H), 7.72 (d, J = 7.6 Hz, 15H), 7.41 (d, J = 7.6 Hz, 15H), 7.20 (s, 4H), 4.76 (s, 4H), 0.71 (s, 24H). ^{31}P{^{1}H}NMR (243 MHz, CD_3CN, 295 K): 5.36 ppm (d, $^2J_{P\text{-}P}$ =21.9 Hz, ^{195}Pt satellites, $^1J_{Pt\text{-}P}$ = 1665.8 Hz), -0.12 ppm (d, $^2J_{P\text{-}P}$ = 21.9 Hz, ^{195}Pt satellites, $^1J_{Pt\text{-}P}$ = 1665.8 Hz). ESI-TOF-MS: *m*/*z* 818.8931 [**Cage 4b** − 8OTf]$^{8+}$, 957.4337 [**Cage 4b** − 7OTf]$^{7+}$, 1141.8347 [**Cage 4b** − 6OTf]$^{6+}$ and 1400.1527 [**Cage 4b** − 5OTf]$^{5+}$.

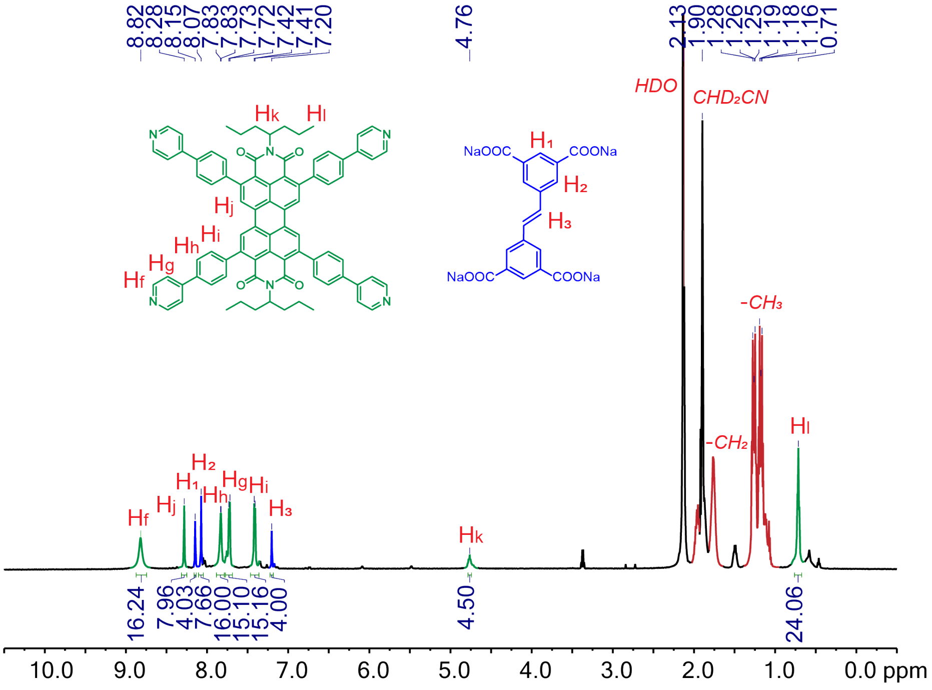

Fig. S8 ^{1}H NMR spectrum (600 MHz, $CDCl_3$, 295 K) recorded for **Cage 4b**

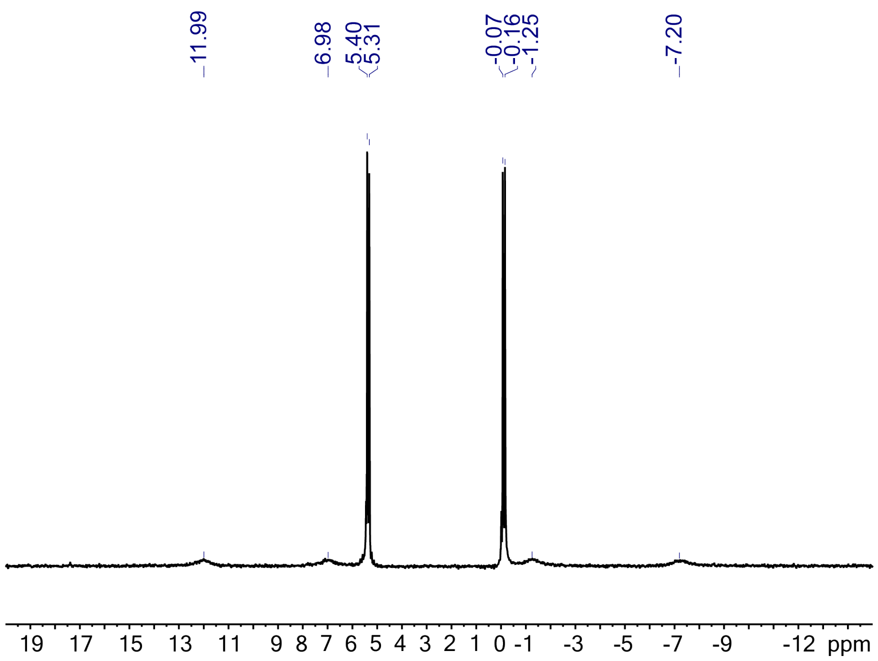

Fig. S9 $^{31}P\{^1H\}$ NMR spectrum (243 MHz, CD_3CN, 295 K) recorded for **Cage 4b**

S3 Crystallographic Data and Refinement Details

Table S1 Crystallographic data and refinement details for **Cage 4a**

Compound	**Cage 4a**
Empirical formula	$C_{256}H_{356}F_{24}N_{12}O_{48}P_{16}Pt_8S_8$
Formula weight	7138.23
Temperature/K	193.00
Crystal system	triclinic
Space group	P^{-1}
a/Å	20.9600(13)
b/Å	22.1707(13)
c/Å	25.1102(16)
α/°	103.154(2)
β/°	109.811(2)
γ/°	98.891(2)
Volume/Å^3	10342.5(11)
Z	1
D_{calc}/g cm^{-3}	1.146
μ/mm^{-1}	2.856
F(000)	3568.0
Theta(max)	25.350
Reflections collected	63132
Independent reflns	36624
R_1;wR_2 [I > 2σ (I)]	0.1099; 0.2220
GOOF	1.083

S4 Host-guest Complexation Study

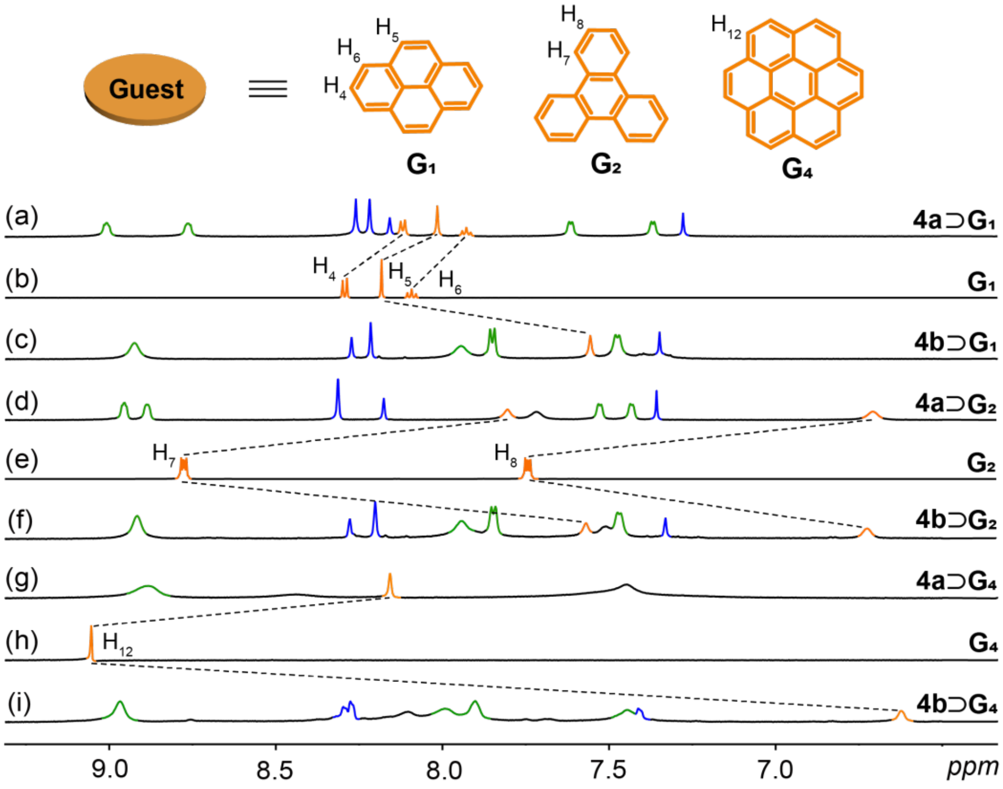

Fig. S10 Partial ^{1}H NMR spectra (CD_3CN) of (**a**) **Cage 4a⊃G1**, (**b**) **G1**, (**c**) **Cage 4b⊃G1**, (**d**) **Cage 4a⊃G2**, (**e**) **G2**, (**f**) **Cage 4b⊃G2**, (**g**) **Cage 4a⊃G4**, (**h**) **G4**, and (**i**) **Cage 4b⊃G4**. [Host] = [Guest]=2 mM

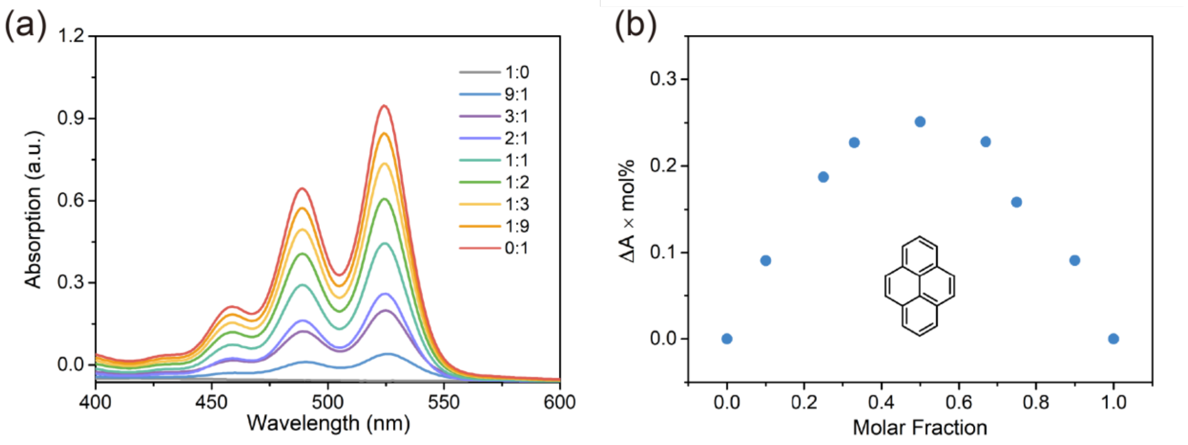

Fig. S11 (**a**) UV-vis absorption of **Cage 4a** with guest **G1** in different molar ratios ([**Cage 4a**] + [**G1**] = 10 μM). (**b**) Job's plot of the complex **Cage 4a⊃G1** in CH_3CN, showing a 1:1 stoichiometry

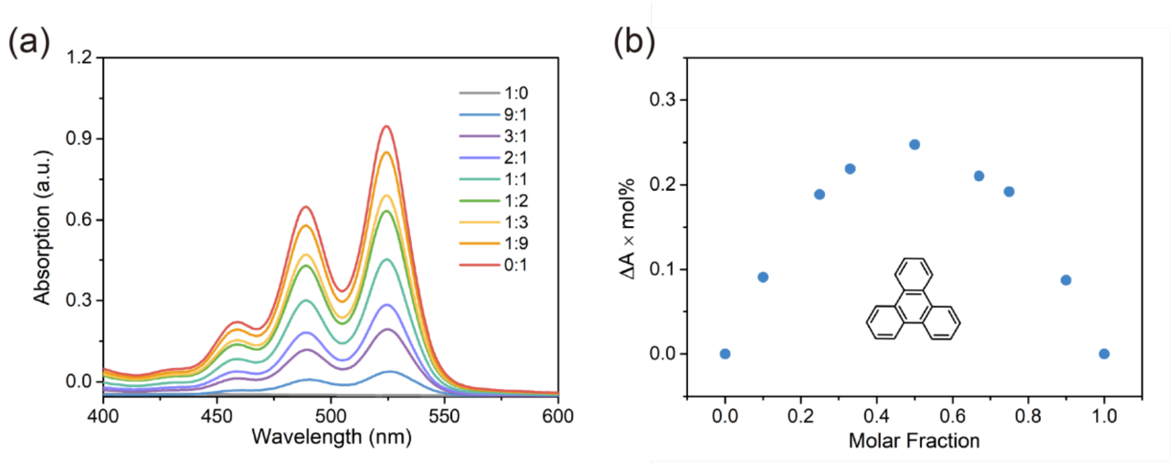

Fig. S12 (**a**) UV-vis absorption of **Cage 4a** with guest **G_2** in different molar ratios ([**Cage 4a**] + [**G_2**] = 10 μM). (**b**) Job's plot of the complex **Cage 4a⊃G_2** in CH_3CN, showing a 1:1 stoichiometry

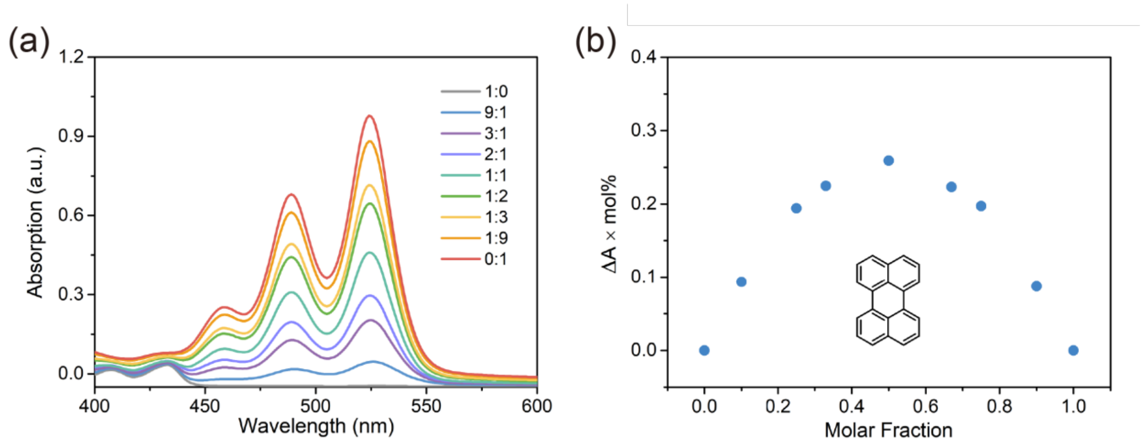

Fig. S13 (**a**) UV-vis absorption of **Cage 4a** with guest **G_3** in different molar ratios ([**Cage 4a**] + [**G_3**] = 10 μM). (**b**) Job's plot of the complex **Cage 4a⊃G_3** in CH_3CN, showing a 1:1 stoichiometry

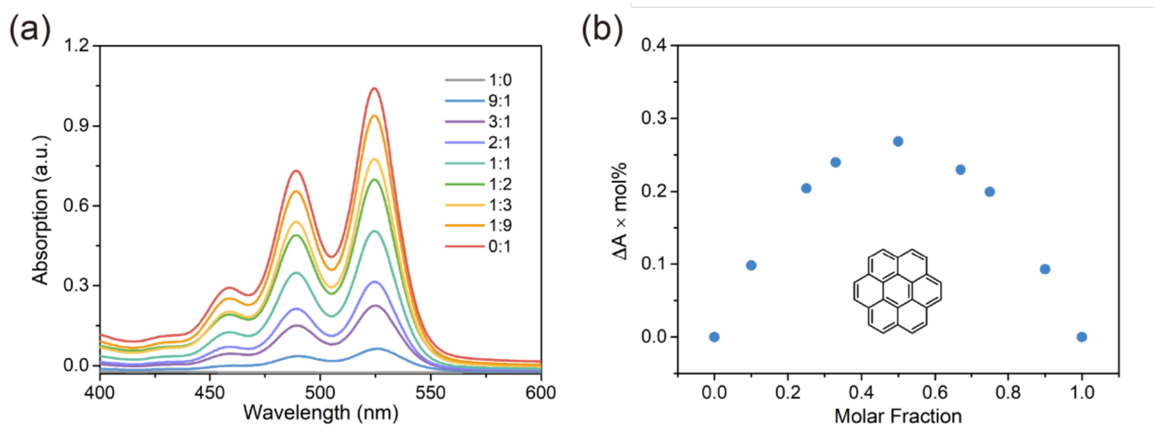

Fig. S14 (**a**) UV-vis absorption of **Cage 4a** with guest **G_4** in different molar ratios ([**Cage 4a**] + [**G_4**] = 10 μM). (**b**) Job's plot of the complex **Cage 4a⊃G_4** in CH_3CN, showing a 1:1 stoichiometry

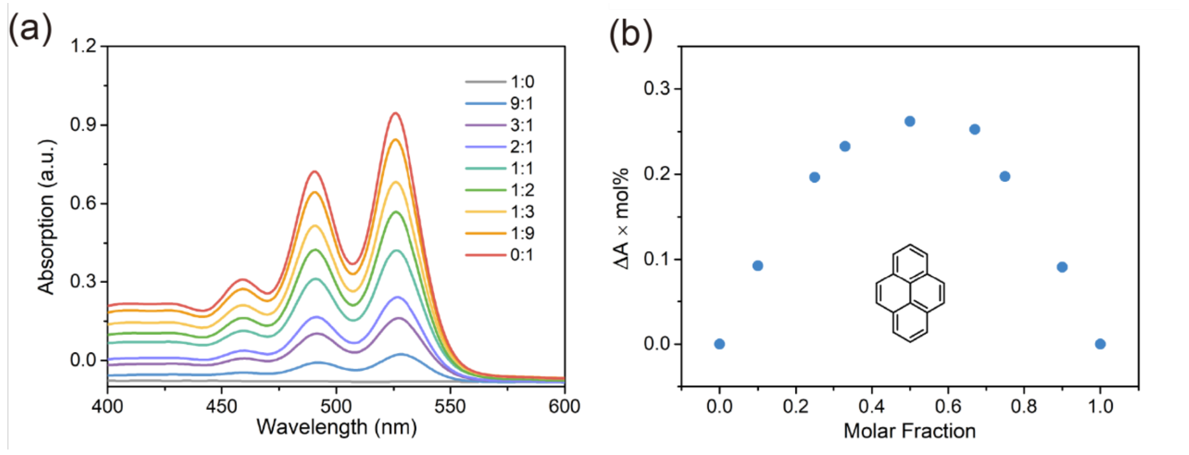

Fig. S15 (**a**) UV-vis absorption of **Cage 4b** with guest $\mathbf{G_1}$ in different molar ratios ([**Cage 4a**] + [$\mathbf{G_1}$] = 10 μM). (**b**) Job's plot of the complex **Cage 4a**$\supset$$\mathbf{G_1}$ in CH_3CN, showing a 1:1 stoichiometry

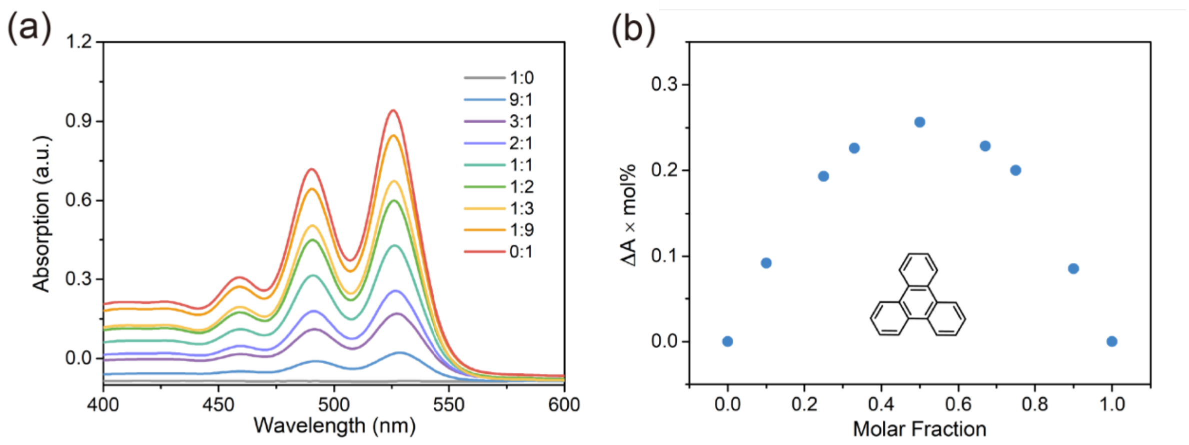

Fig. S16 (**a**) UV-vis absorption of **Cage 4b** with guest $\mathbf{G_2}$ in different molar ratios ([**Cage 4b**] + [$\mathbf{G_2}$] = 10 μM). (**b**) Job's plot of the complex **Cage 4b**$\supset$$\mathbf{G_2}$ in CH_3CN, showing a 1:1 stoichiometry

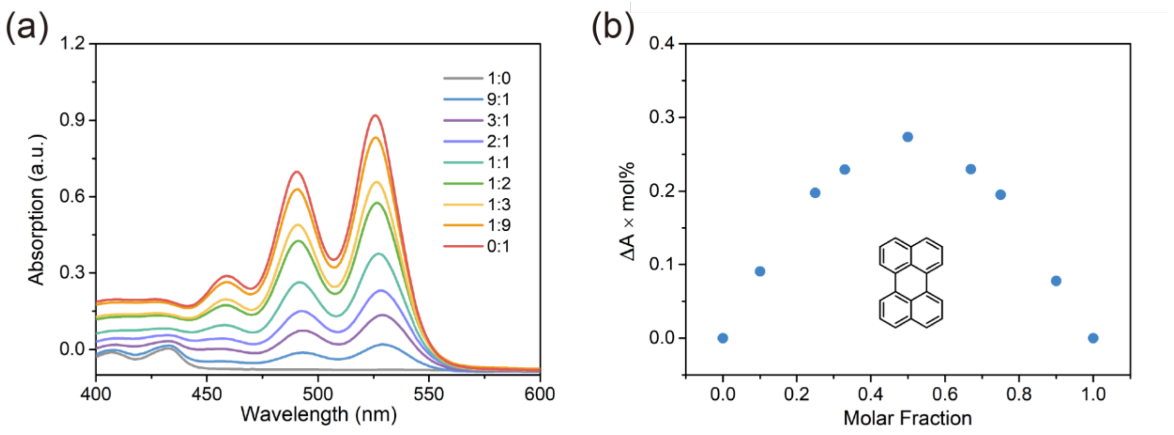

Fig. S17 (**a**) UV-vis absorption of **Cage 4b** with guest $\mathbf{G_3}$ in different molar ratios ([**Cage 4b**] + [$\mathbf{G_3}$] = 10 μM). (**b**) Job's plot of the complex **Cage 4b**$\supset$$\mathbf{G_3}$ in CH_3CN, showing a 1:1 stoichiometry

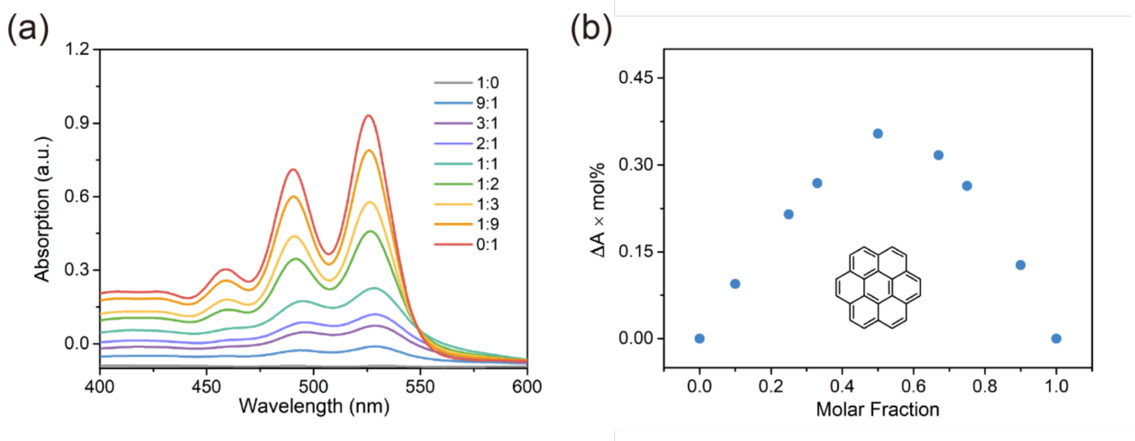

Fig. S18 (**a**) UV-vis absorption of **Cage 4b** with guest **G_4** in different molar ratios ([**Cage 4b**] + [**G_4**] = 10 μM). (**b**) Job's plot of the complex **Cage 4b⊃G_4** in CH_3CN, showing a 1:1 stoichiometry

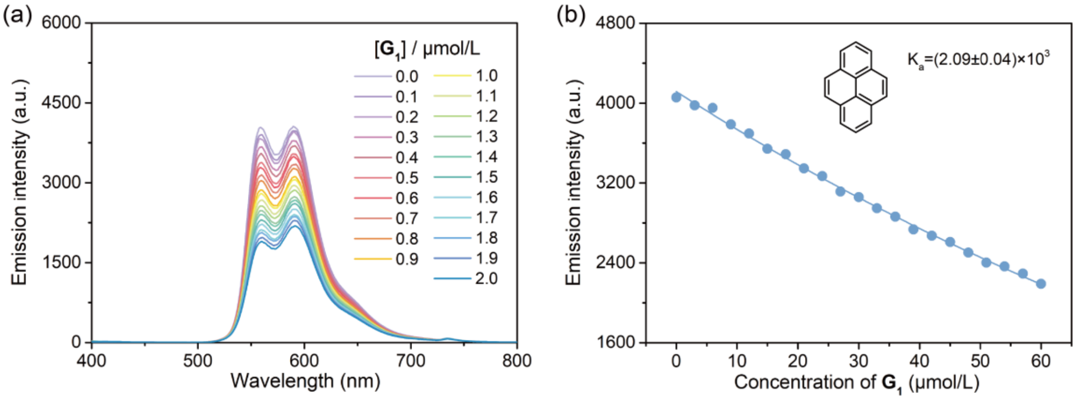

Fig. S19 (**a**) Fluorescence spectra of **Cage 4a** at a fixed concentration upon the addition of **G_1** in CH_3CN ([Host] = 10 μM, [Guest] = 500 μM). (**b**) Nonlinear fitting curves of the emission intensity at 365 nm of **Cage 4a** versus the concentrations of **G_1**

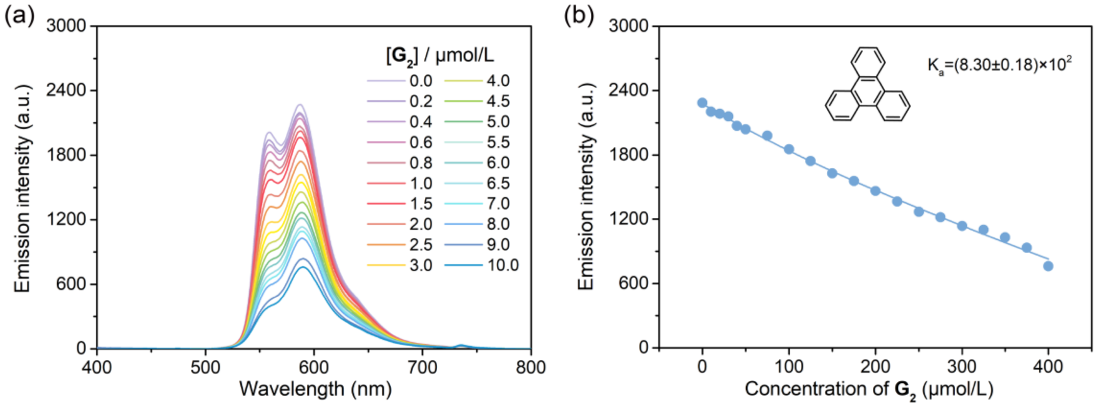

Fig. S20 (**a**) Fluorescence spectra of **Cage 4a** at a fixed concentration upon the addition of **G_2** in CH_3CN ([Host] = 10 μM, [Guest] = 500 μM). (**b**) Nonlinear fitting curves of the emission intensity at 365 nm of **Cage 4a** versus the concentrations of **G_2**

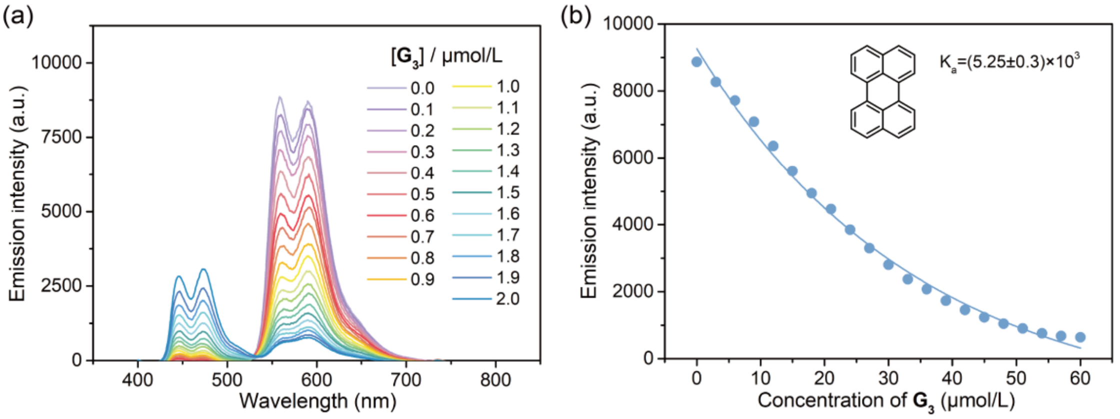

Fig. S21 (**a**) Fluorescence spectra of **Cage 4a** at a fixed concentration upon the addition of **G**$_3$ in CH_3CN ([Host] = 10 μM, [Guest] = 500 μM). (**b**) Nonlinear fitting curves of the emission intensity at 365 nm of **Cage 4a** versus the concentrations of **G**$_3$

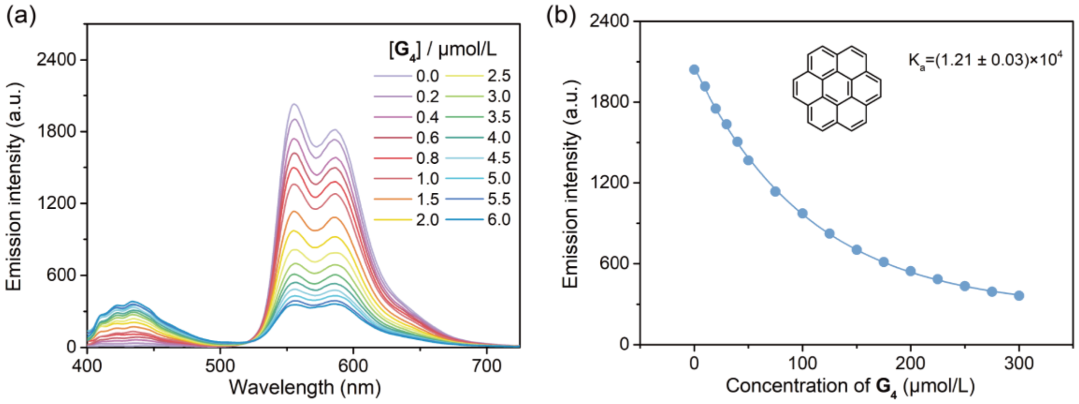

Fig. S22 (**a**) Fluorescence spectra of **Cage 4a** at a fixed concentration upon the addition of **G**$_4$ in CH_3CN ([Host] = 10 μM, [Guest] = 500 μM). (**b**) Nonlinear fitting curves of the emission intensity at 365 nm of **Cage 4a** versus the concentrations of **G**$_4$

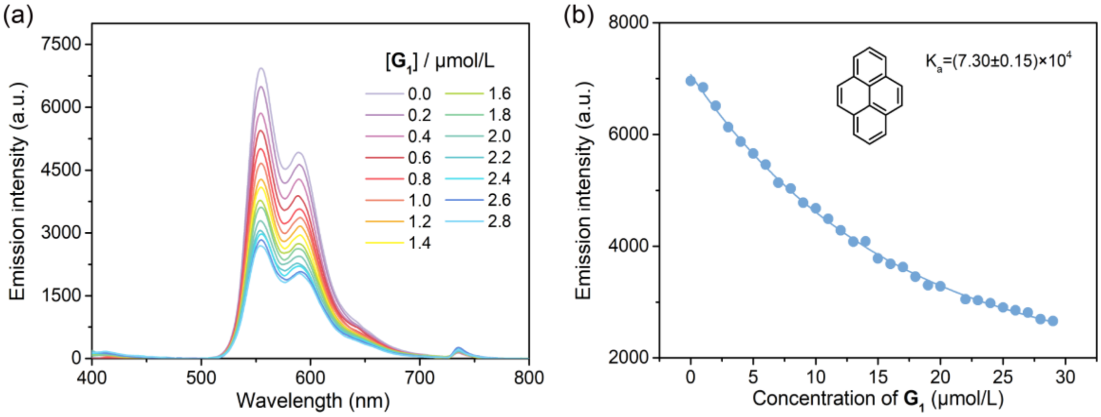

Fig. S23 (**a**) Fluorescence spectra of **Cage 4b** at a fixed concentration upon the addition of **G**$_1$ in CH_3CN ([Host] = 10 μM, [Guest] = 200 μM). (**b**) Nonlinear fitting curves of the emission intensity at 365 nm of **Cage 4b** versus the concentrations of **G**$_1$

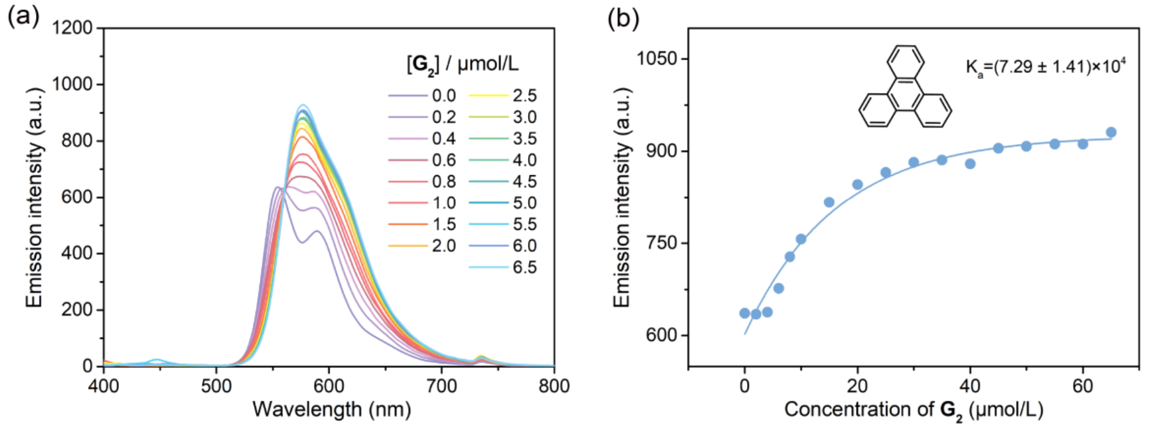

Fig. S24 (**a**) Fluorescence spectra of **Cage 4b** at a fixed concentration upon the addition of **G2** in CH_3CN ([Host] = 10 μM, [Guest] = 200 μM). (**b**) Nonlinear fitting curves of the emission intensity at 365 nm of **Cage 4b** versus the concentrations of **G2**

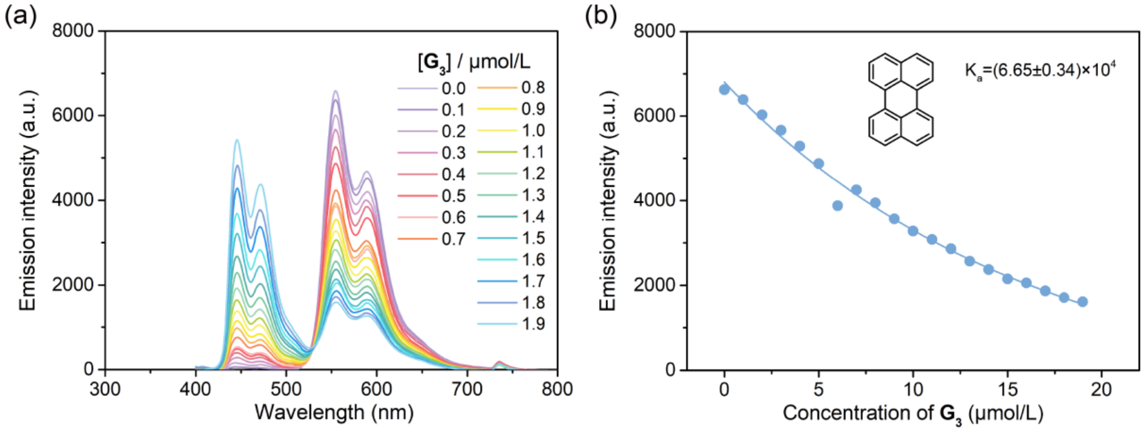

Fig. S25 (**a**) Fluorescence spectra of **Cage 4b** at a fixed concentration upon the addition of **G3** in CH_3CN ([Host] = 10 μM, [Guest] = 200 μM). (**b**) Nonlinear fitting curves of the emission intensity at 365 nm of **Cage 4b** versus the concentrations of **G3**

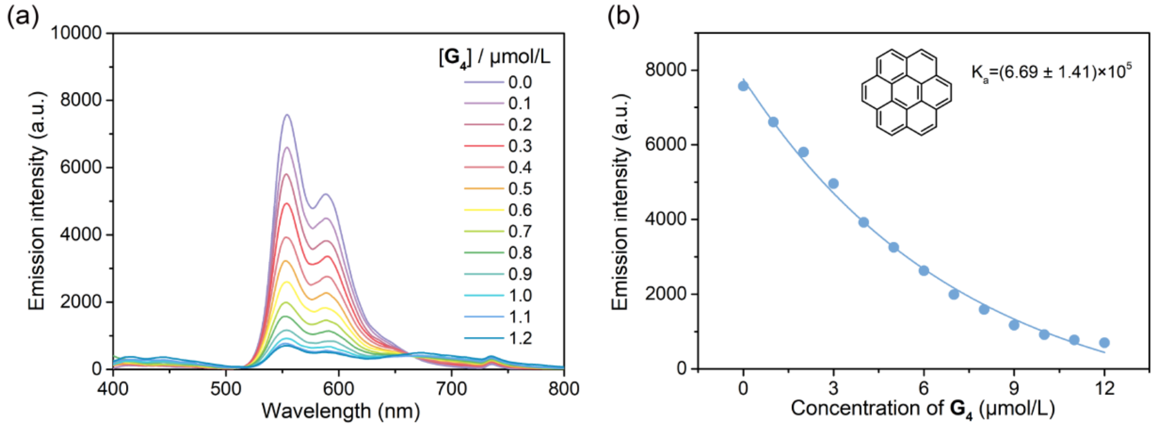

Fig. S26 (**a**) Fluorescence spectra of **Cage 4b** at a fixed concentration upon the addition of **G4** in CH_3CN ([Host] = 10 μM, [Guest] = 200 μM). (**b**) Nonlinear fitting curves of the emission intensity at 365 nm of **Cage 4b** versus the concentrations of **G4**

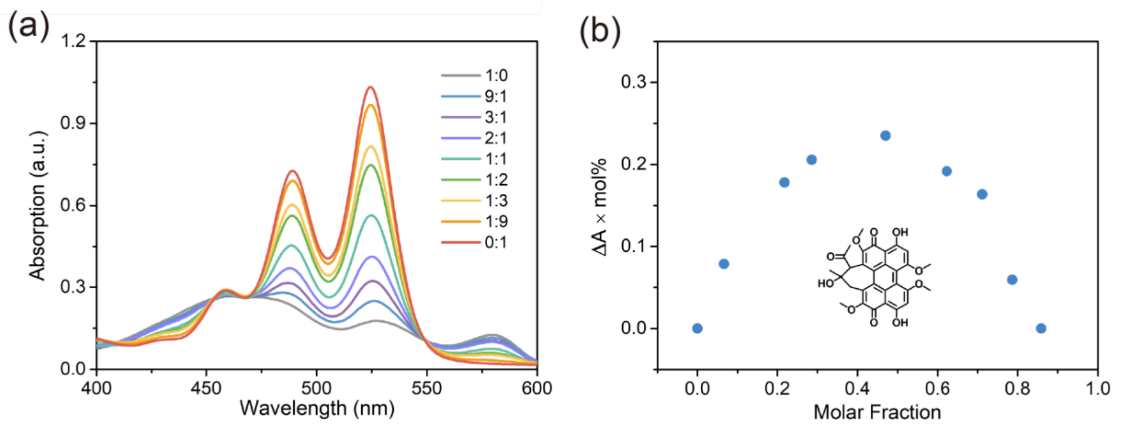

Fig. S27 (**a**) UV-vis absorption of **Cage 4a** with guest **G_5** in different molar ratios ([**Cage 4a**] + [**G_5**] = 10 μM). (**b**) Job's plot of the complex **Cage 4a⊃G_5** in CH_3CN, showing a 1:1 stoichiometry

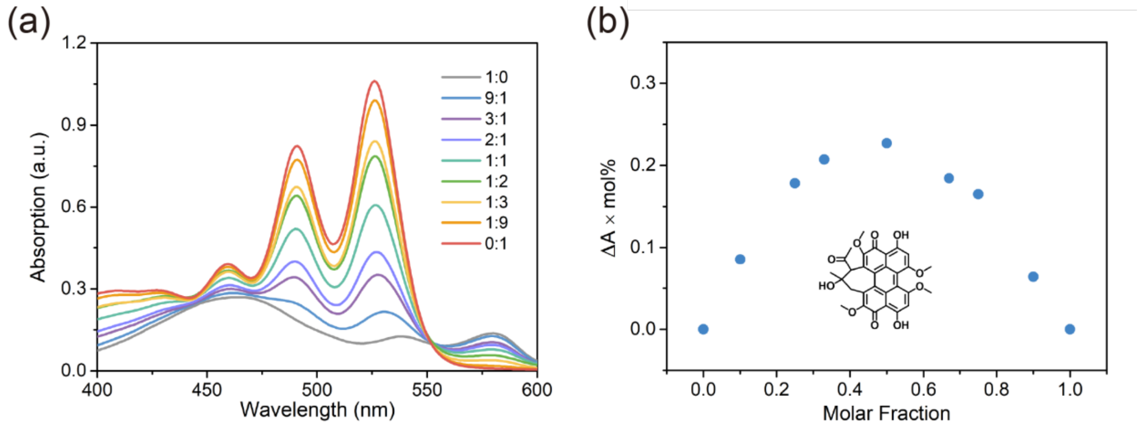

Fig. S28 (**a**) UV-vis absorption of **Cage 4b** with guest **G_5** in different molar ratios ([**Cage 4b**] + [**G_5**] = 10 μM). (**b**) Job's plot of the complex **Cage 4b⊃G_5** in CH_3CN, showing a 1:1 stoichiometry

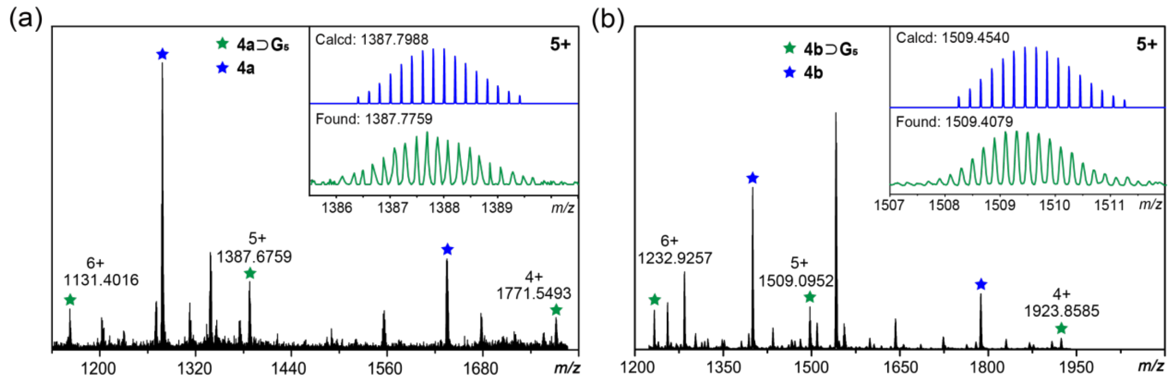

Fig. S29 ESI-TOF-MS spectra of (**a**) **Cage 4a⊃G_5** and (**b**) **Cage 4b⊃G_5**

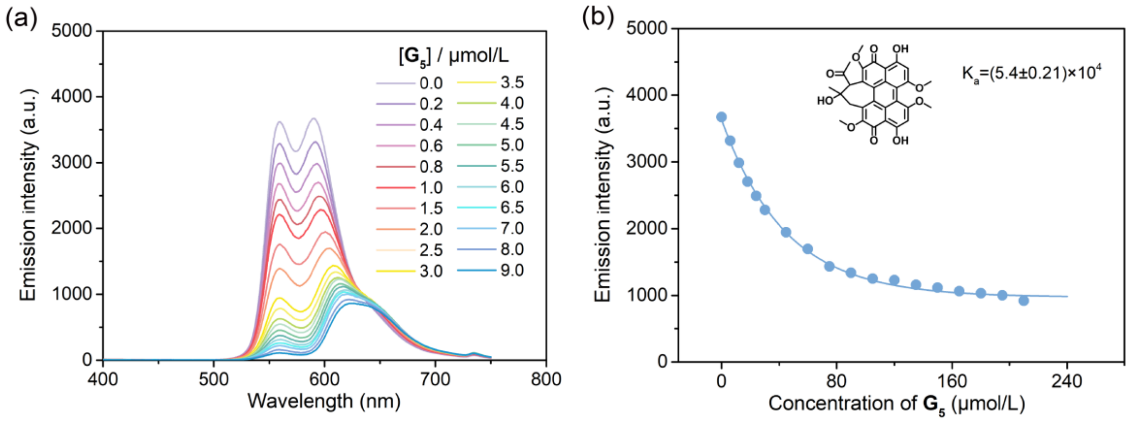

Fig. S30 (**a**) Fluorescence spectra of **Cage 4a** at a fixed concentration upon the addition of **G5** in CH_3CN ([Host] = 10 μM, [Guest] = 500 μM). (**b**) Nonlinear fitting curves of the emission intensity at 365 nm of **Cage 4a** versus the concentrations of **G5**

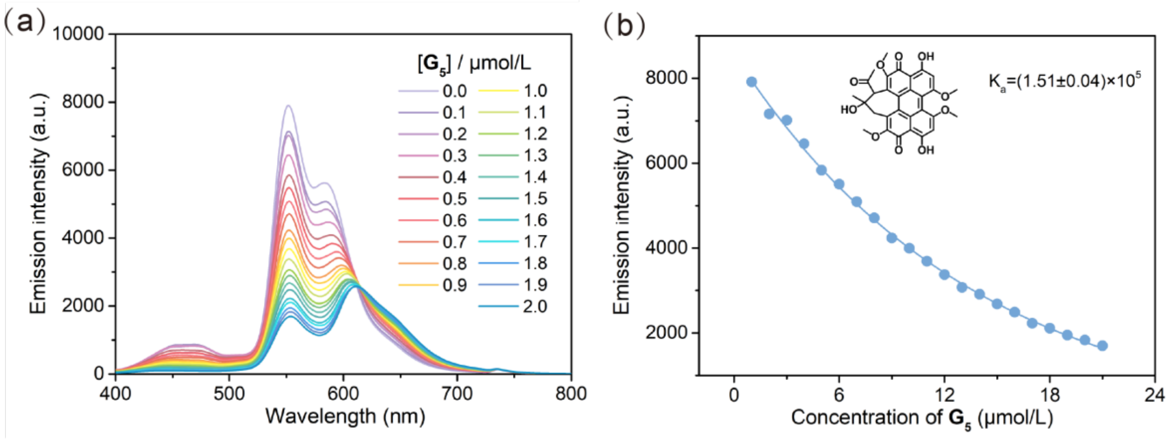

Fig. S31 (**a**) Fluorescence spectra of **Cage 4b** at a fixed concentration upon the addition of **G5** in CH_3CN ([Host] = 10 μM, [Guest] = 200 μM). (**b**) Nonlinear fitting curves of the emission intensity at 365 nm of **Cage 4b** versus the concentrations of **G5**

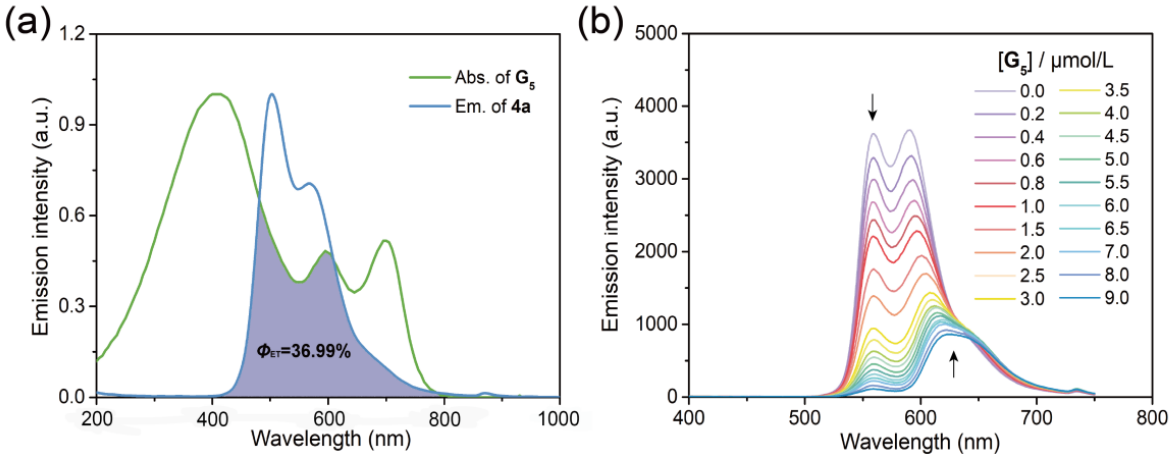

Fig. S32 (**a**) Normalized absorption and emission spectra of compounds **G5** and **Cage 4a**. (**b**) Fluorescence spectra of **Cage 4a** (c = 10.0 μM) and **G5** (c = 500.0 μM) in a mixture of CH_3CN and water (v/v = 1/1) with different concentrations of **G5**

S5 Measurements of Singlet Oxygen

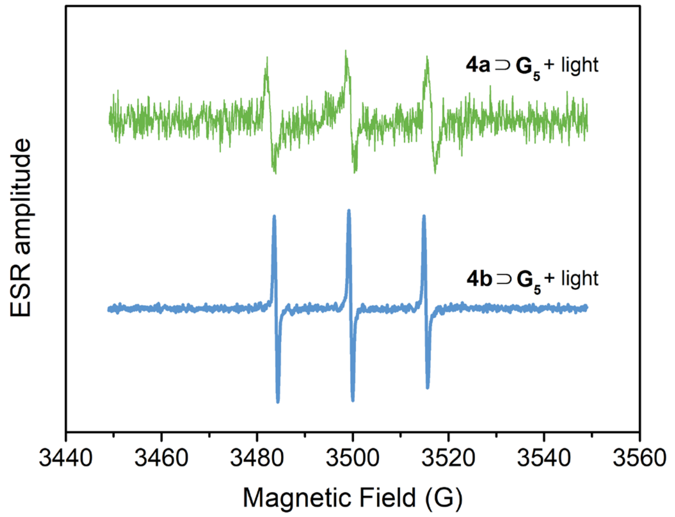

Fig. S33 ESR spectra of complexes **Cage 4a⊃G$_5$** and **Cage 4b⊃G$_5$** under illumination (white light, 100 mW/cm^2), using TEMP as a spin trap agent

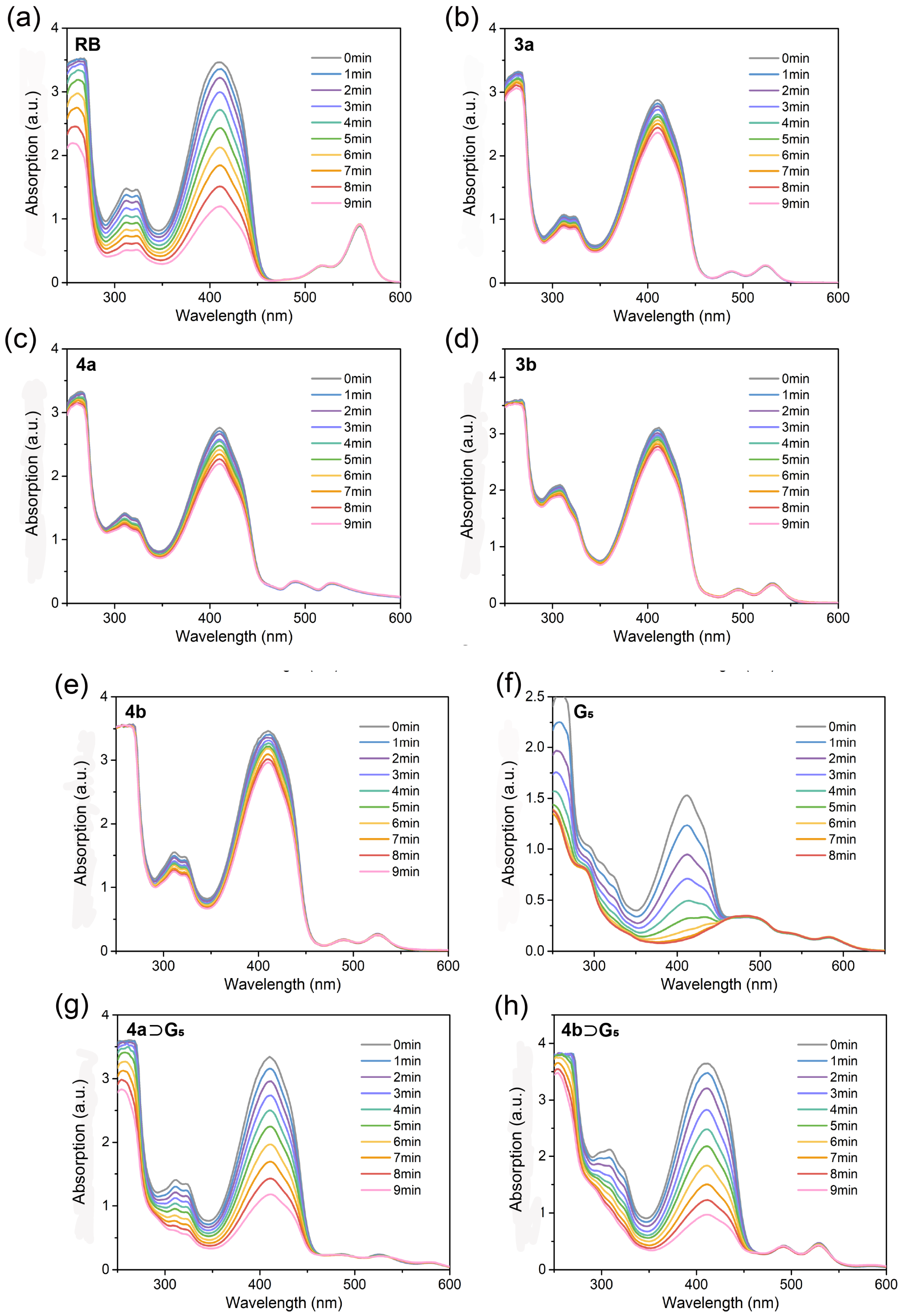

Fig. S34 The absorption decays of DPBF at 410 nm vs irradiation time in the presence of (**a**) **RB**, (**b**) **3a**, (**c**) **Cage 4a**, (**d**) **3b**, (**e**) **Cage 4b**, (**f**) **G$_5$**, (**g**) **Cage 4a⊃G$_5$** and (h) **Cage 4b⊃G$_5$** upon irradiation in acetonitrile (λ_{ex} = 520 nm)

S6 Photophysical Property

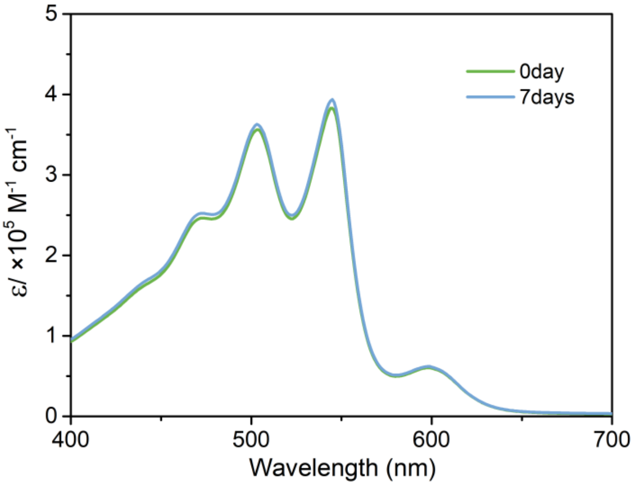

Fig. S35 UV−vis absorption of **NPs 5** in 1% DMSO/H_2O within 7 days

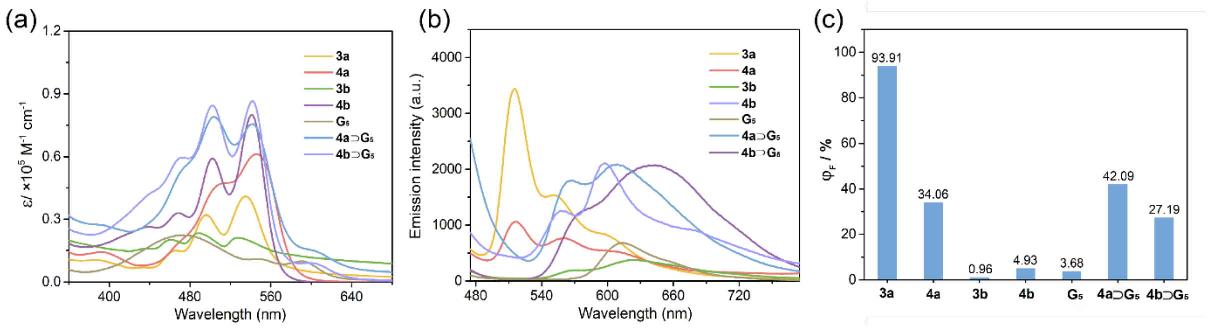

Fig. S36 (**a**) UV−vis absorption, (**b**) emission spectra and (**c**) fluorescence quantum yield of **3a**, **Cage 4a**, **3b**, **Cage 4b**, **G$_5$**, **Cage 4a⊃G$_5$** and **Cage 4b⊃G$_5$** in 1% DMSO/H_2O. Excitation wavelengths: λ_{ex} = 400 nm

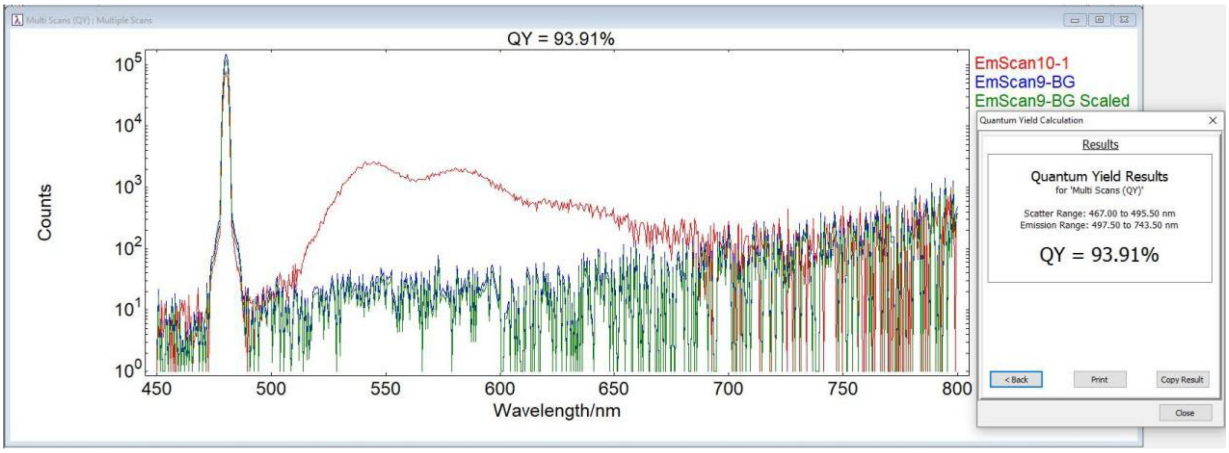

Fig. S37 Absolute fluorescence quantum yield of ligand **3a**

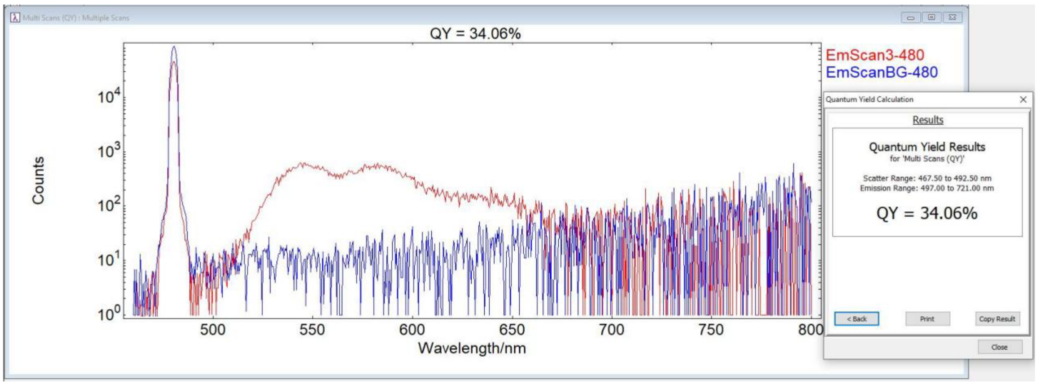

Fig. S38 Absolute fluorescence quantum yield of **Cage 4a**

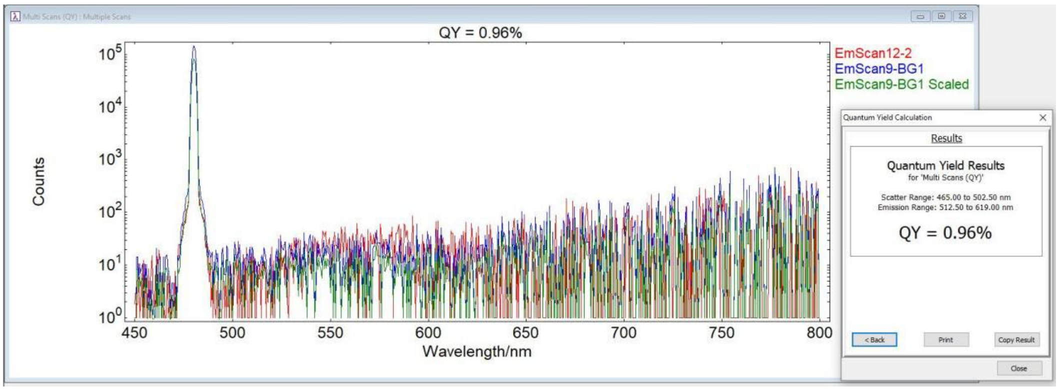

Fig. S39 Absolute fluorescence quantum yield of ligand **3b**

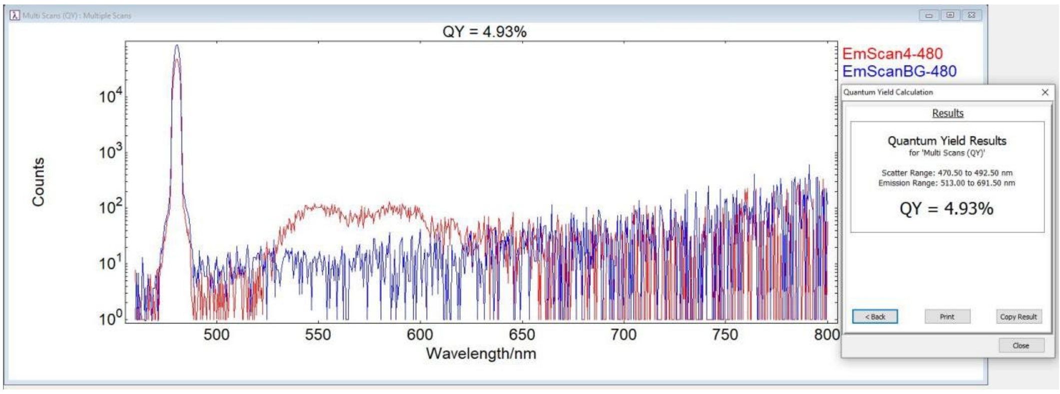

Fig. S40 Absolute fluorescence quantum yield of **Cage 4b**

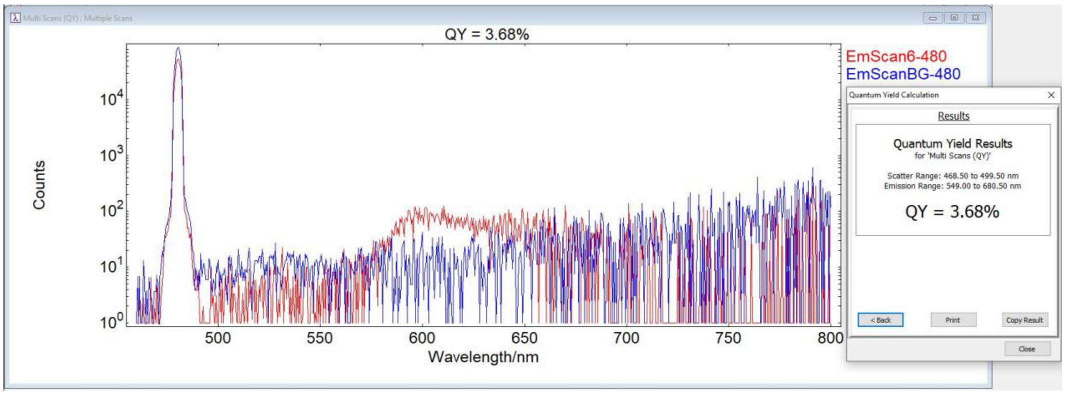

Fig. S41 Absolute fluorescence quantum yield of **G_5**

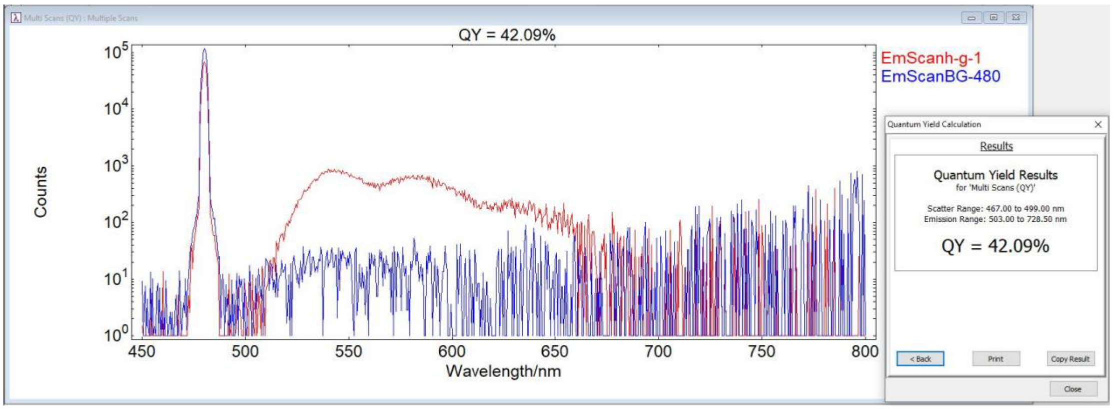

Fig. S42 Absolute fluorescence quantum yield of **Cage 4a⊃G$_5$**

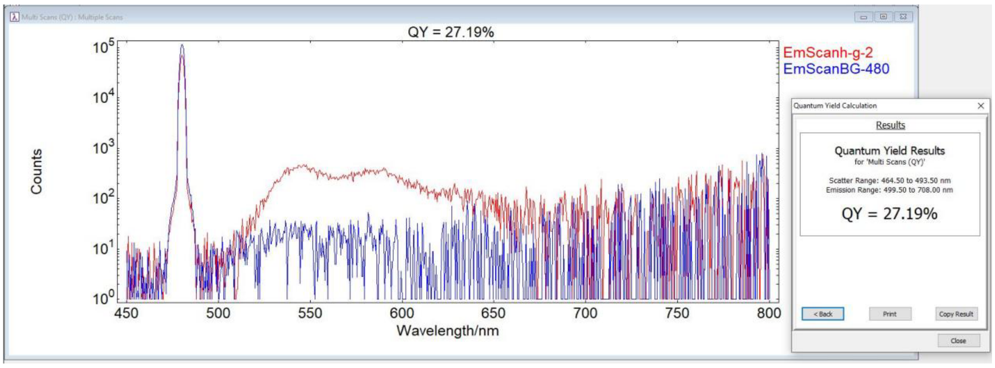

Fig. S43 Absolute fluorescence quantum yield of **Cage 4b⊃G$_5$**

S7 Fluorescence Intensity of Different Compounds at Different Time

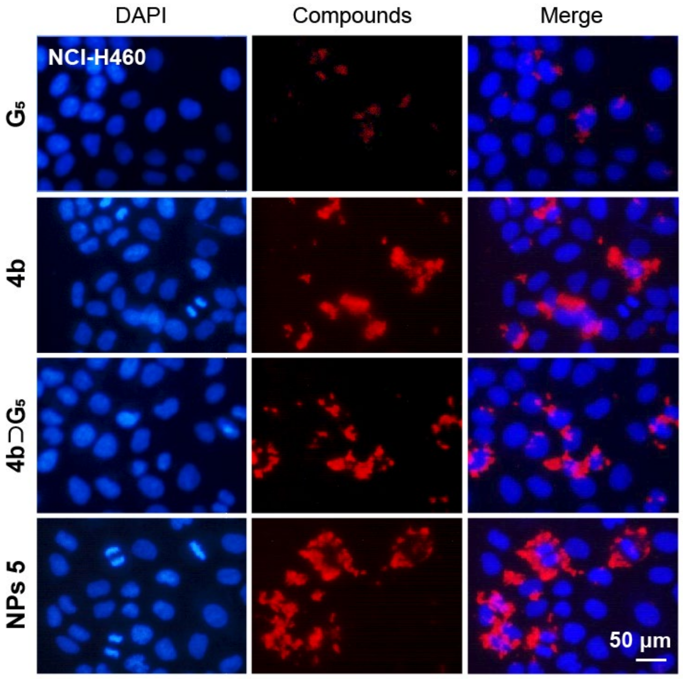

Fig. S44 CLSM images of NCI-H460 cells after the incubation with DAPI and **G$_5$**, **Cage 4b**, **Cage 4b⊃G$_5$** or **NPs 5**

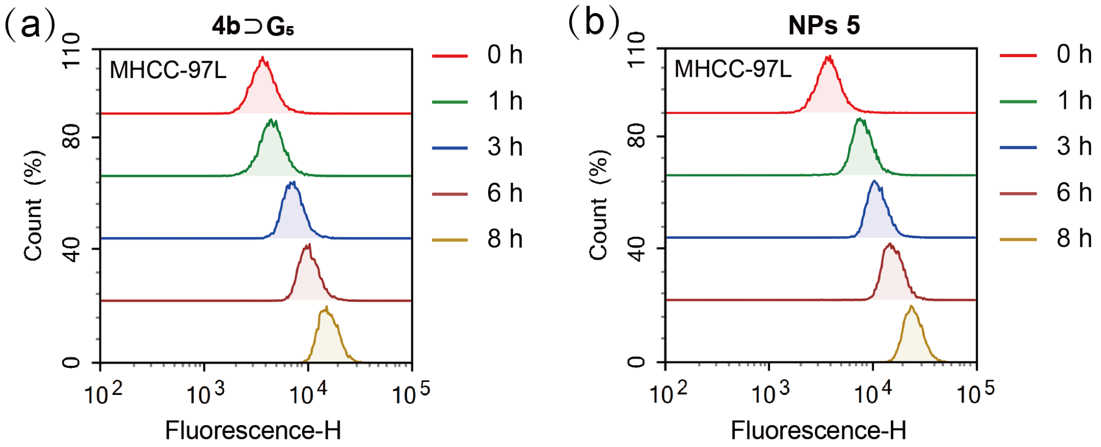

Fig. S45 Flow cytometric analysis of MHCC-97L cells with emission of **Cage 4b⊃G5** (**a**) or **NPs 5** (**b**) at different incubation times

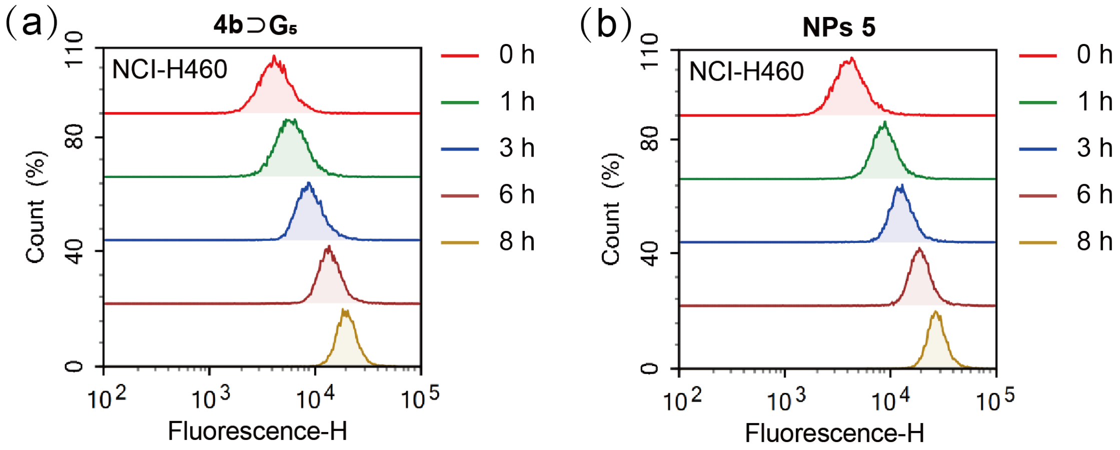

Fig. S46 Flow cytometric analysis of NCI-H460 cells with emission of **Cage 4b⊃G5** (**a**) or **NPs 5** (**b**) at different incubation times

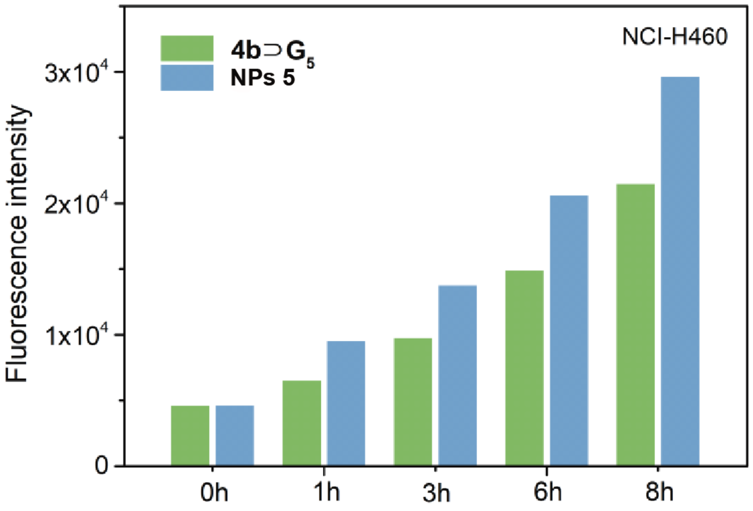

Fig. S47 Fluorescence intensity of NCI-H460 cells incubated with **Cage 4b⊃G5** or **NPs 5** at different times

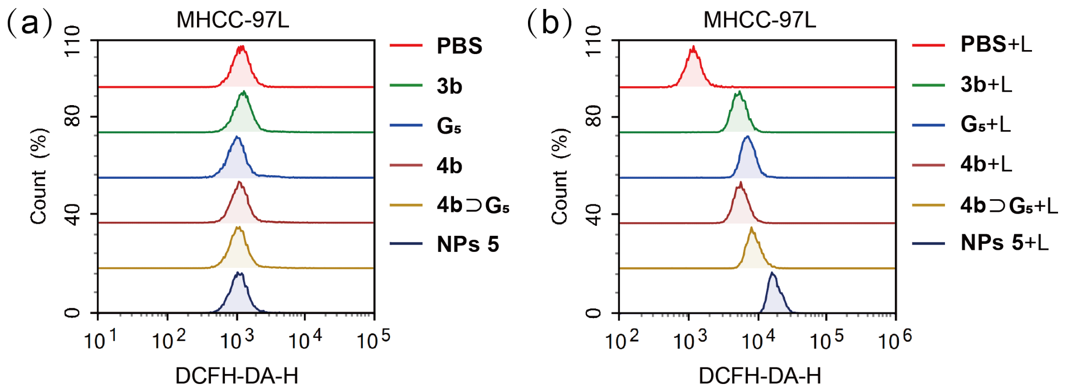

Fig. S48 The fluorescence signal of MHCC-97L cells treated with PBS, **3b**, **G**$_5$, **Cage 4b**, **Cage 4b⊃G**$_5$ and **NPs 5** without light (**a**) or with light (**b**)

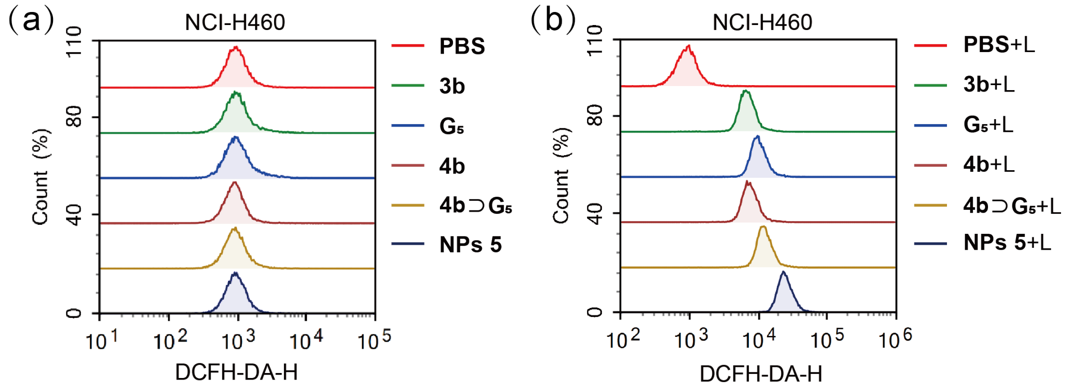

Fig. S49 The fluorescence signal of NCI-H460 cells treated with PBS, **3b**, **G**$_5$, **Cage 4b**, **Cage 4b⊃G**$_5$ and **NPs 5** without light (**a**) or with light (**b**)

S8 MTT Assay of Different Compounds against Cancer Cells

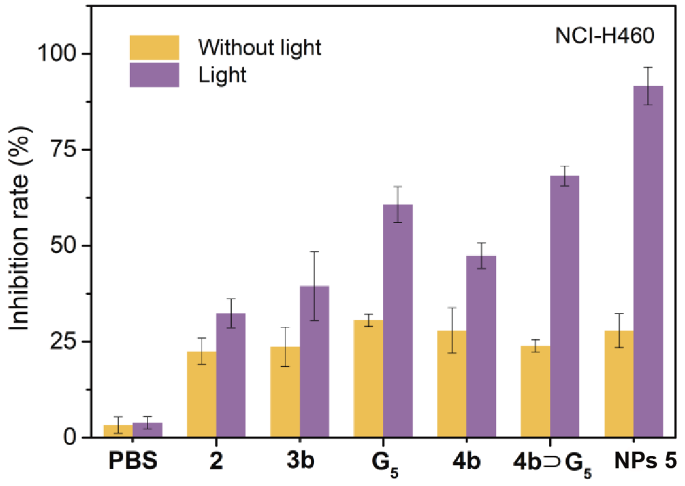

Fig. S50 MTT assay of NCI-H460 cells after the incubation with **3b**, **G**$_5$, **Cage 4b**, **Cage 4b⊃G**$_5$ and **NPs 5**

S9 In vivo Tumor Therapy Study

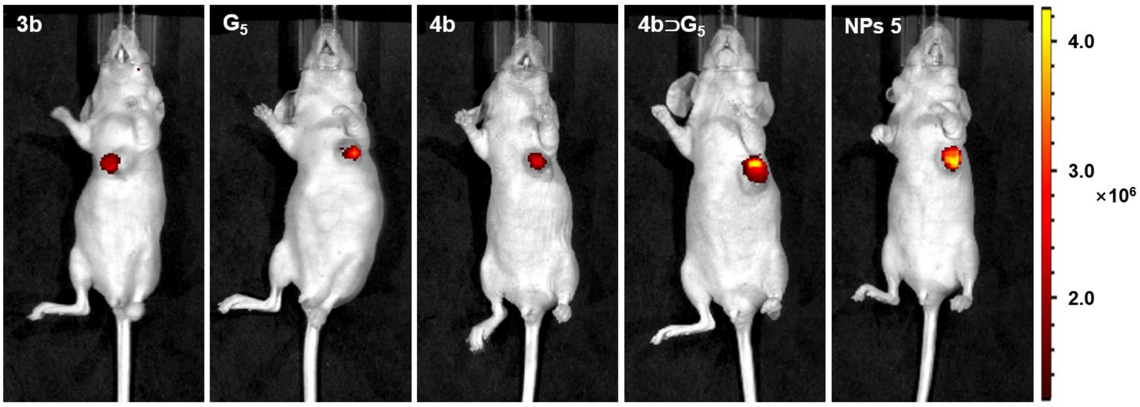

Fig. S51 In vivo fluorescence imaging of the tumor-bearing mice after injection of **2**, **3b**, **G_5**, **Cage 4b**, **Cage 4b⊃G_5** and **NPs 5**

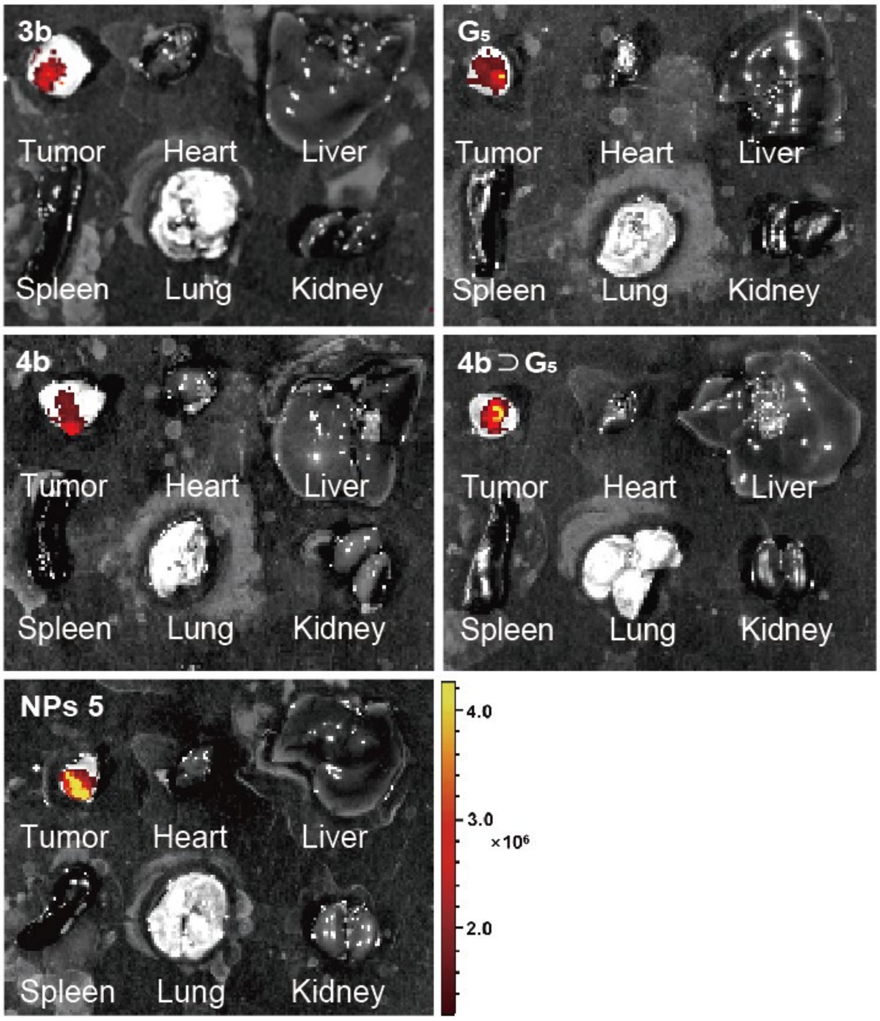

Fig. S52 Images of different organs after injection of **2**, **3b**, **G_5**, **Cage 4b**, **Cage 4b⊃G_5** and **NPs 5**

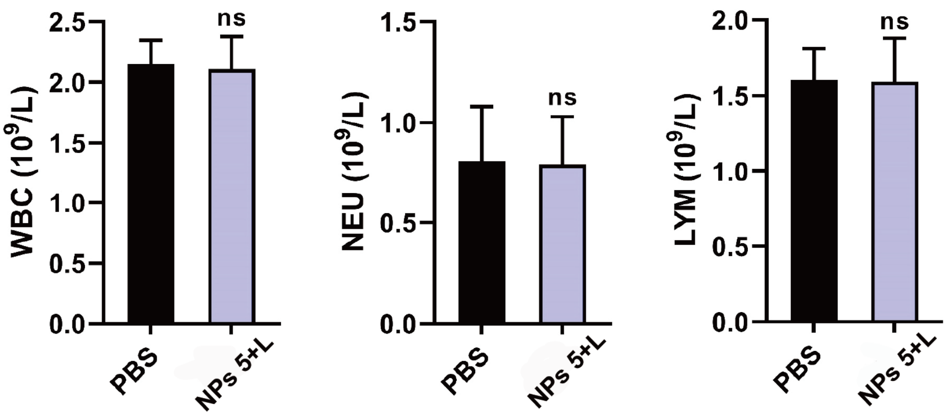

Fig. S53 Hematology analysis of the tumor-bearing mice with PBS or **NPs 5** + L. No significant changes with all tested parameters were detected. White blood cells: WBC, neutrophil: NEU, lymphocyte: LYM

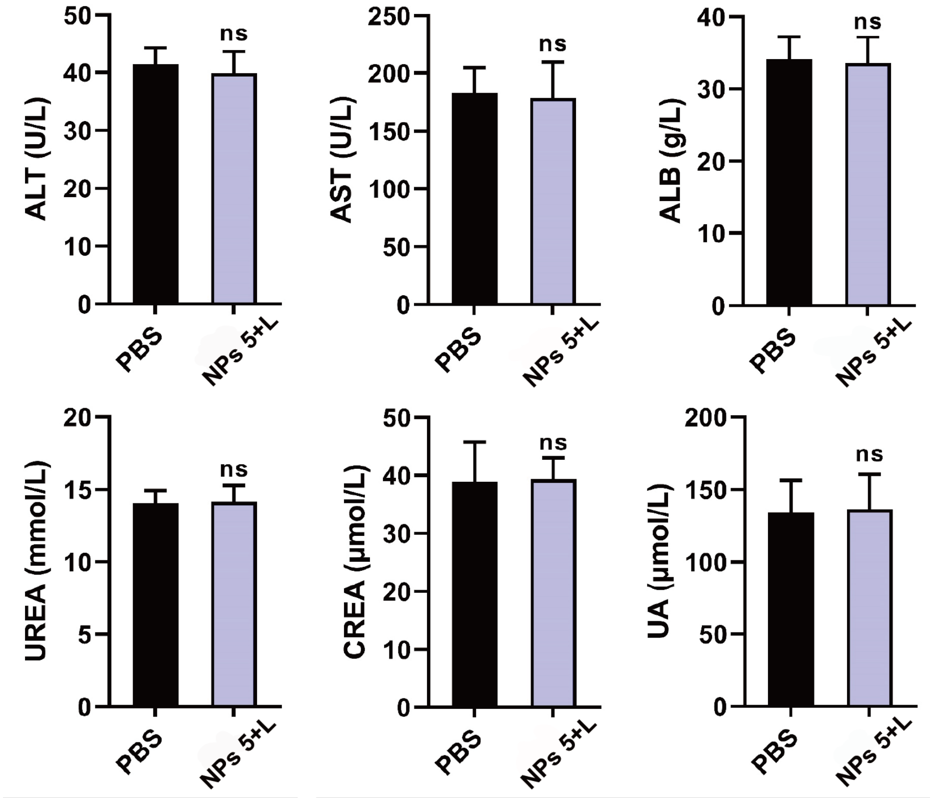

Fig. S54 Alterations of alanine transaminase (ALT), aspartate transaminase (AST), albumin (ALB), urea nitrogen (UREA), creatinine (CREA), and uric acid (UA) in the tumor-bearing mice (n = 5 for each group) with PBS or **NPs 5** + L

Supplementary References

[S1]Y. Zheng, Z. Zhao, M. Wang, K. Ghosh, J.B. Pollock et al., A facile approach toward multicomponent supramolecular structures: selective self-assembly via charge separation. J. Am. Chem. Soc. **2010**, *132*, 16873. https://doi.org/10.1021/ja106251f

[S2]T. Teraoka, S. Hiroto, H. Shinokubo, Iridium-catalyzed direct tetraborylation of perylene bisimides. Org. Lett. **2011**, 13, 2532. https://doi.org/10.1021/ol2004534

[S3]Y. Hou, Z. Zhang, S. Lu, J. Yuan, Q. Zhu et al., Highly emissive perylene diimide-based metallacages and their host–guest chemistry for information encryption. J. Am. Chem. Soc. **2020**, 142, 18763. https://doi.org/10.1021/jacs.0c09904
